# The hidden cost of antibiotics: microbial dysbiosis-induced SCFA imbalance and its underlying mechanisms

**DOI:** 10.3389/fimmu.2026.1917541

**Published:** 2026-09-07

**Authors:** Sona R. Ayvazyan, Olga V. Darvina, Klavdiya A. Turkadze, Sergey G. Gerasimov, Nina N. Kanshina

**Affiliations:** Department of Infectious Diseases, Institute of Public Health named after F.F. Erisman, Sechenov First Moscow State Medical University (Sechenov University), Moscow, Russia

**Keywords:** antibiotic-induced dysbiosis, barrier integrity, FFAR2/FFAR3 signaling, gut–brain axis, immunomodulation, microbiota restoration, SCFA imbalance, Tregs

## Abstract

Antibiotics, especially broad-spectrum types, disrupt the gut microbiota by indiscriminately killing both pathogenic and beneficial bacteria, leading to dysbiosis. This dysbiosis directly contributes to an imbalance in metabolites, such as short-chain fatty acids (SCFA) produced by gut bacteria from indigestible carbohydrates. Such shifts promote the selective expansion of facultative anaerobic pathogens, particularly Proteobacteria, thereby exacerbating epithelial oxidative stress and low-grade inflammation. Simultaneously, reduced SCFA availability disrupts tight junction expression, increases intestinal permeability, and impairs immune regulation by limiting Regulatory T cell (Treg) differentiation and attenuating G protein-coupled receptor (GPCR) (e.g., Free fatty acid receptor (FFAR) 2/3) and histone deacetylase (HDAC)-dependent anti-inflammatory pathways. Furthermore, antibiotic-induced SCFA depletion impairs GPCR-mediated signaling and disrupts vagal and enteroendocrine pathways that mediate gut–brain communication. Consequences of this antibiotic-dysbiosis-SCFA imbalance cascade include short-term issues like antibiotic-associated diarrhea (AAD), as well as long-term risks such as obesity, allergies, asthma, inflammatory diseases, and even impacts on brain development in neonates. In this review, we overview the mechanistic insights of how antibiotics reshape microbiota-derived SCFA profiles and highlight the implications for host health and disease, ultimately underscoring the need for targeted strategies such as microbiota restoration and prebiotic supplementation to mitigate these hidden consequences.

## 1 Introduction

Antibiotics represent one of the most revolutionary achievements in the history of medicine; however, their effectiveness has increasingly been undermined in recent decades due to the rapid emergence and global spread of antibiotic-resistant bacterial strains (1). Beyond resistance, growing evidence shows that antibiotics can profoundly disrupt the composition and function of the gut microbiota (2, 3). The gut microbiota, dominated under healthy conditions by Firmicutes and Bacteroidetes, plays essential roles in nutrient metabolism, maintenance of mucosal integrity, immune homeostasis, and the production of short-chain fatty acids (SCFAs), primarily acetate, propionate, and butyrate (4, 5). These metabolites serve as important regulators of epithelial energy supply, inflammatory signaling, metabolic balance, and gut-brain communication (6). Antibiotic-induced dysbiosis profoundly alters the microbial communities responsible for SCFA production, disrupting both their synthesis and downstream metabolic functions (7, 8). Indeed, such disturbances can have far-reaching consequences for host physiology. As an example, mice treated with broad-spectrum antibiotics, including vancomycin, ciprofloxacin, and metronidazole, suffered a marked decrease in the intestinal concentration of the three main SCFAs (acetate, propionate, and butyrate) (9). Firmicutes and Bacteroidetes represent the dominant phyla in the healthy gut microbiota, where they contribute to mucosal barrier integrity, immune function modulation, and the production of key SCFAs (10). This balance is easily disrupted by factors such as antibiotic exposure, reduced dietary fiber intake, or ongoing intestinal inflammation, creating conditions that favor the overgrowth of opportunistic taxa like Enterobacteriaceae (11). Such dysbiosis compromises epithelial barrier function, enabling microbial products, including lipopolysaccharide (LPS), to translocate into the circulation and trigger systemic inflammatory responses (12, 13). Of note, the parallel depletion of SCFA-producing bacteria limits the signals required for regulatory T (Treg) cell differentiation, thereby further amplifying inflammation (10).

SCFAs are quickly absorbed in the colon, where they provide the major energy source for colonocytes and impact cell functions that include proliferation, differentiation, and apoptosis (14, 15). Reduced SCFA levels are directly associated with metabolic disorders, including obesity, and with gastrointestinal diseases, including inflammatory bowel disease (IBD) (16). Antibiotic-induced metabolic disturbances extend beyond the gut in manners that alter vagal pathways involved in gut–brain communication, disrupt lipid metabolism, influence vascular calcification mechanisms, and impair enteroendocrine activity and glucose regulation (16). This review covers the various mechanisms by which antibiotics interfere with the microbiota–SCFA axis and details the affected cellular and molecular pathways and ensuing health and disease implications of these interrelations. Understanding such interrelations can guide efforts toward developing targeted interventions—such as probiotics, prebiotics, high-fiber dietary strategies, and direct SCFA supplementation—that can mitigate the often-overlooked adverse effects of antibiotic therapy.

## 2 Microbiota-derived SCFA function in health and disease

**Figure 1:**
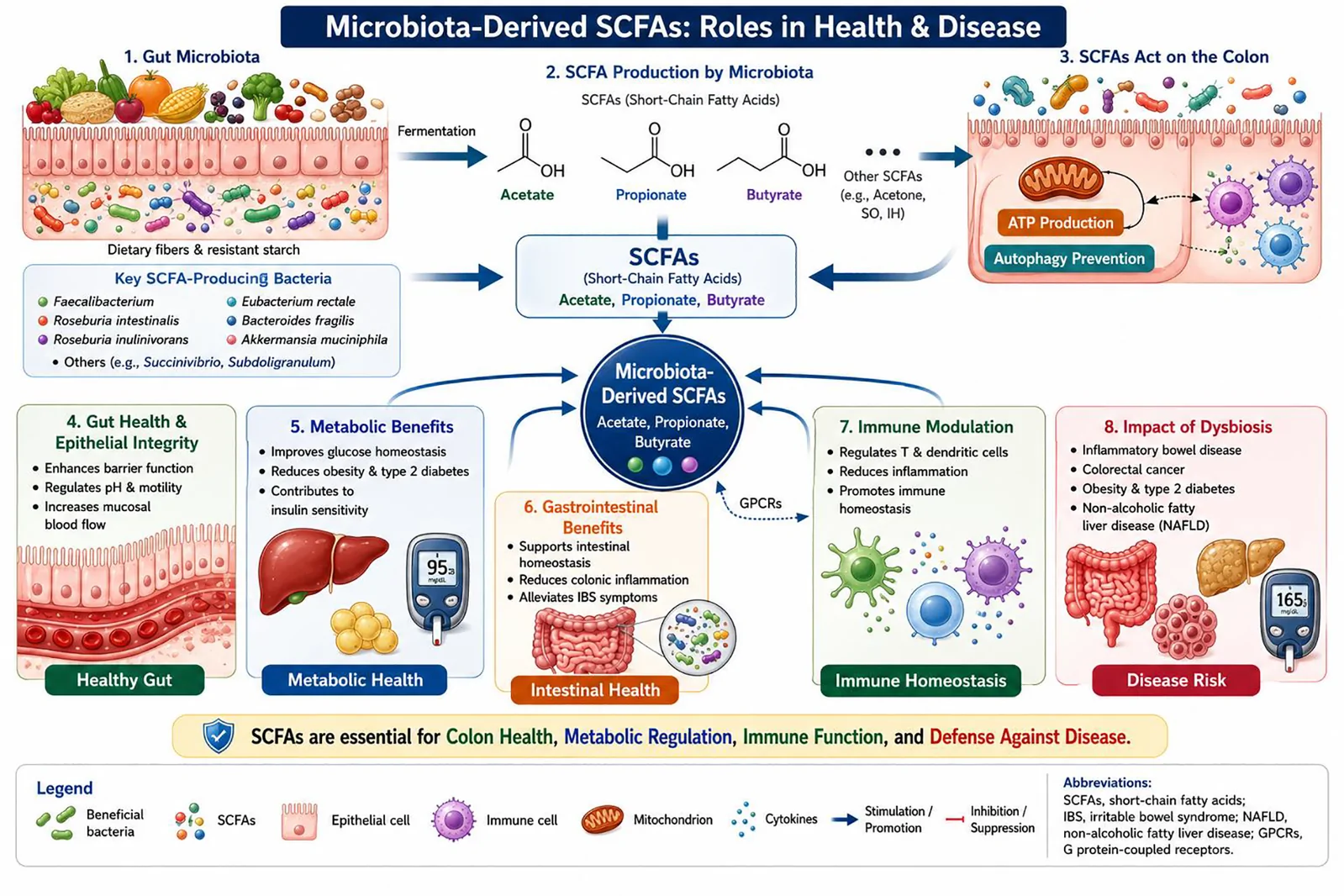
Microbiota-derived short-chain fatty acids (SCFAs) generation and roles in health and disease. Dietary fibers and resistant starches that escape digestion in the upper gastrointestinal tract are fermented by gut microbiota, including members of the genera Faecalibacterium, Roseburia, Eubacterium, Bacteroides, Akkermansia, and Subdoligranulum, leading to the production of the major SCFAs: acetate, propionate, and butyrate. Following their production in the colon, SCFAs are absorbed by colonocytes and serve as important energy substrates, particularly butyrate, which supports mitochondrial ATP production, epithelial barrier integrity, and cellular homeostasis. Microbiota-derived SCFAs exert pleiotropic effects on host physiology. In the intestinal epithelium, SCFAs strengthen barrier function, regulate luminal pH and gut motility, and enhance mucosal blood flow. Systemically, SCFAs improve glucose homeostasis, promote insulin sensitivity, regulate lipid metabolism, and contribute to metabolic health. Through activation of G protein-coupled receptors (GPCRs), including free fatty acid receptor 2 (FFAR2/GPR43) and free fatty acid receptor 3 (FFAR3/GPR41), SCFAs modulate immune responses by regulating T cells (Tregs), dendritic cells (DCs), cytokine production, and immune homeostasis. SCFAs also support gastrointestinal health by maintaining intestinal homeostasis and reducing inflammatory responses. Conversely, disruption of the gut microbiota (dysbiosis) reduces SCFA production and is associated with increased susceptibility to inflammatory bowel disease (IBD), colorectal cancer, obesity, type 2 diabetes mellitus (T2DM), and non-alcoholic fatty liver disease (NAFLD). Collectively, microbiota-derived SCFAs function as key mediators of host–microbiome interactions and contribute to intestinal, metabolic, and immune health.

SCFAs are important contributors to colonic health and epithelial integrity (**Figure 1**) (17). Among them, butyrate is the principal metabolic fuel for colonocytes, providing about 60–70% of the energy required for growth and differentiation of these cells (18). In line with this notion, colonocytes from germ-free mice-deprived of microbiota-derived SCFAs, exhibit severe energy deficiency, reflected in the lower expression of mitochondrial enzymes responsible for fatty acid metabolism (19). Consequently, these cells present defective mitochondrial respiration, characterized by a lower Nicotinamide adenine dinucleotide (NADH)/NAD^+^ ratio, attenuated production of ATP, and lowered oxidative phosphorylation, rendering these cells susceptible to autophagy. Notably, exogenously added butyrate rescues these metabolic defects when provided to isolated colonocytes from germ-free mice (20). Beyond their role as an important energy substrate, SCFAs have numerous physiological effects in the gut, including the modulation of colonic motility, mucosal blood flow, and luminal pH, thus affecting the absorption of electrolytes and nutrients (19). Many of these effects are believed to be mediated through the activation of G protein-coupled receptors (GPCRs) (17). Importantly, the impact of SCFAs extends well beyond local epithelial and digestive functions; they also have important immunomodulatory functions both within the gut and at systemic levels (21).

SCFAs are closely associated with the development and progression of metabolic disorders, inflammatory diseases, and cancer (22). Dysbiosis of the intestinal microbiota is a hallmark of IBD, and SCFA-producing species like Faecalibacterium prausnitzii and Roseburia intestinalis are significantly depleted in affected individuals (23–25). Their loss disrupts critical interactions between commensal bacteria and host immune cells. Accordingly, levels of acetate and propionate are significantly decreased in the intestinal lumen of patients with IBD (26). Since SCFAs modulate the development, function, and migration of both innate and adaptive immune cells, they represent important immunomodulatory agents with beneficial effects in the context of IBD (27). In Colorectal cancer (CRC), there has been a reported increase in the abundance of Escherichia coli and Bacteroides fragilis, while the usual SCFA-producing bacteria like Akkermansia muciniphila are found to be significantly reduced in numbers (28, 29). SCFAs result from microbial fermentation of dietary fiber, and butyrate acts as a major energy source for the intestinal epithelial cells (IECs). This butyrate suppresses the initiation and progression of colon cancer through the regulation of tumor suppressor gene expression, and increases apoptosis (30).

The gut microbiota and SCFAs also have a central role in controlling host metabolic processes, and microbial dysbiosis is considered a major driving force for common metabolic disorders (31). In mice, soluble fiber-derived propionate and butyrate stimulate intestinal gluconeogenesis (IGN) through complementary mechanisms (32). IGN contributes to glucose and energy homeostasis, improves metabolic status, and contributes to the control of body weight and glycaemia. Butyrate stimulates IGN gene expression through a Cyclic AMP (cAMP)-dependent mechanism, whereas propionate, as an IGN substrate, promotes the transcription of IGN genes by activating the gut–brain neural circuit (32). SCFAs thus exert a stimulatory effect on IGN to protect against metabolic disease in murine models (33). Most current research into gut microbiota-derived metabolites in metabolic disorders has targeted obesity, type 2 diabetes (T2D), and Non-Alcoholic Fatty Liver Disease (NAFLD), whereas relatively few studies have investigated thyroid dysfunction or hyperuricemia (34–36).

## 3 Effect of antibiotics on the microbiota

Although antibiotics target the elimination of specific pathogenic bacteria, many of the agents in clinical use have broad-spectrum activity and are thus able to treat a wide range of infections (37). Consequently, these drugs commonly impact non-target members of the gut microbiota, and changes in community structure commonly persist well beyond the cessation of treatment (38, 39). Furthermore, antibiotic exposure can select for the overgrowth of resistant strains, thereby establishing a resident reservoir of resistance genes in the gut (40). Treatment with antibiotics commonly results in a marked reduction in microbial diversity (38). Despite this disruption, the gut microbiota generally exhibits substantial resilience, with overall community profiles returning toward their original composition, often within days to weeks (41, 42). However, full recovery is often incomplete: whereas many taxa re-establish their niches, other microbial groups do not rebound and may be lost from the community permanently (38, 39).

Of note, antibiotic use during pregnancy, infancy, and childhood has consistently been associated with altered neonatal adiposity and heightened risk of allergies, asthma, and a number of metabolic disorders across childhood (43–45). Due to the increased susceptibility of newborns, particularly premature infants, to infection, antibiotics are among the most commonly prescribed classes of medications (46). The more frequently utilized ones include amoxicillin, co-amoxiclav, benzylpenicillin, cephalosporins, gentamicin, vancomycin, clindamycin, and azithromycin (47). Early antibiotic therapy, although lifesaving, severely depletes gut microbial diversity and causes significant changes in the bacterial composition of the gut, the most prominent feature of which includes a decrease in beneficial taxa such as Bifidobacterium (48). Prolonged use in preterm infants has resulted in profound microbial changes characterized by low diversity of bacterial populations, leading to an increase in the rate of Necrotizing enterocolitis (NEC) (49).

Experimental work using murine models has further demonstrated that commonly administered antibiotics, such as ampicillin, vancomycin, metronidazole, and neomycin, cause immediate and long-lasting changes in the gut microbiome (50). Short-term changes include depleted microbial diversity and elimination of important commensals, such as Bifidobacterium. After the administration of antibiotics, the microbiota may take several weeks to months to partially recover, during which time the host is more susceptible to opportunistic infection and the emergence of antibiotic-resistant organisms. Other short-term complications include antibiotic-associated diarrhea (AAD) and Clostridium difficile-associated disease (CDAD) (51). AAD is usually caused by disruption of normal microbial flora consisting of bacteria such as Klebsiella pneumoniae, *Staphylococcus aureus*, and CDAD, resulting from the overgrowth of C. difficile, which can cause recurrent disease and life-threatening colitis (51).

Perturbations in the early microbiota result in durable changes in community structure, an increased expression and persistence of antibiotic-resistance genes, and a heightened incidence of obesity, allergic disease, asthma, and IBD later in life (52). These findings are supported by reports that gut colonization starts *in utero* due to placental factors and is continuously shaped by mode of delivery, type of infant feeding, and other early environmental exposures (2, 53). Disruption of this process due to antimicrobial interventions thus has enduring effects on microbial ecology, contributing to poor outcomes from both metabolic and immune standpoints. Antibiotic therapy can have prolonged effects on microbial communities, even in adults. For instance, studies in healthy adults administered cefprozil, ciprofloxacin, or amoxicillin show that changes in microbiota composition may persist 12 weeks after treatment, during which time microbiota composition would only partially recover and continue to harbor resistant strains (41, 54). More notably, antibiotics with shorter half-life periods, such as clindamycin, induce more pronounced disruptions, often persisting for two years after exposure (38, 40).

## 4 Methodology for the literature review

The present review was conducted to comprehensively summarize the current evidence regarding antibiotic-induced alterations in SCFA homeostasis and the underlying mechanisms linking these changes to host physiology and disease. To ensure broad coverage of the available literature, a systematic bibliographic search was performed using the Web of Science, PubMed, and Google Scholar databases. The search primarily focused on publications investigating the effects of antibiotics on the gut microbiota, microbial SCFA production, host immune responses, intestinal barrier function, metabolism, and disease outcomes. The primary search keywords included (“antibiotic” AND “SCFA”), (“antibiotic” AND “short-chain fatty acid”), (“antibiotic” AND “butyrate”), (“antibiotic” AND “propionate”), (“antibiotic” AND “acetate”), (“antibiotic” AND “gut microbiota”), (“antibiotic” AND “microbial dysbiosis”), and related combinations. To provide a comprehensive overview, additional searches were performed for individual antibiotic classes, including β-lactams, glycopeptides, aminoglycosides, macrolides, tetracyclines, fluoroquinolones, lincosamides, polymyxins, sulfonamides, nitroimidazoles, oxazolidinones, rifamycins, and carbapenems, among others, in combination with SCFA-related terms. Reference lists of relevant publications were also manually examined to identify additional studies that may not have been retrieved during the initial database search. No restrictions were imposed on the year of publication to capture both foundational studies and the most recent advances in the field. Articles published in English were preferentially included, and both preclinical and clinical investigations were considered. The screening process involved an initial evaluation of titles and abstracts to determine relevance, followed by a detailed assessment of the full text of potentially eligible studies.

## 5 Antibiotic-induced SCFA imbalance and its underlying mechanisms

In this section, we review the mechanistic evidence detailing how antibiotic exposure disrupts the microbiota–SCFA axis and discuss the downstream implications for function in host cells (**Table 1**).

**Table 1:** Antibiotic-induced imbalance in microbiota-derived SCFA and its mechanisms.

Antibiotic type	Study setting	Exposure time	Dose of antibiotics	Metabolomic method	Affected bacteria/microbiota diversity	SCFAs impacted	Function of SCFAs	Result of imbalance	Reference
Broad-spectrum antibiotics (ampicillin,metronidazole, neomycin, gentamicin, and vancomycin)	*In vivo* (Mouse)	7 days	Ampicillin (1g/liter),metronidazole (1g/liter), neomycin (1g/liter), gentamicin (1g/liter),and vancomycin (0.5g/liter)	GC-MS	Increased ratio of Bacteroidetes/Firmicutes, Allobaculum genus(Firmicutes phylum)	Butyrate, acetate, propionate	SCFAs maintain macrophage hyporesponsiveness, regulate metabolic activity (increase OxPhos), promote alternative macrophage activation, and support intestinal immune tolerance	Antibiotics induced hyperresponsive intestinal macrophages producing excess inflammatory cytokines. Re-exposure to a normal microbiota failed to restore immune tolerance, resulting in prolonged TH1 inflammation, increased susceptibility to Citrobacter rodentium and Trichuris muris, and persistent dysbiosis. SCFA depletion was a key driver; butyrate supplementation restored macrophage hyporesponsiveness, corrected T cell dysfunction, and re-established immune homeostasis.	(55)
Antibiotic cocktail consisted of ampicillin, kanamycin, metronidazole, and vancomycin)	*In vivo* (Mouse) model + *in vitro* human macrophages	7 days	1 g/Lampicillin (1 g/L kanamycin (K4000); 0.5 g/L metronidazole(Sigma, M3761); 0.5 g/L vancomycin)	–	Loss of butyrate-producingClostridia species	Butyrate (primary), other SCFAs reduced as part of microbiota loss	Butyrate enhances macrophage efferocytosis capacity, supports transcriptional programs controlling apoptotic cell clearance, and maintains TIM-4 expression on LPMs	Antibiotic treatment or germ-free conditions caused impaired efferocytosis in large peritoneal macrophages due to depletion of microbiota-derived butyrate. Butyrate supplementation restored efferocytosis efficiency, normalized TIM-4 expression, and reversed macrophage defects. Efferocytosis remained impaired even after stopping antibiotics, contributing to worsened autoimmune disease in a lupus model.	(56)
Broad-spectrum prenatal antibiotics (maternal exposure)	*In vivo* (murine model; pregnant dams and neonatal offspring)	During pregnancy (prenatal exposure)	Not specified (broad-spectrum cocktail administered to dams)	GC-MS	Depletion of maternal and neonatal gut microbiota; reduction in SCFA-producing bacteria, including butyrate-producing Firmicutes	↓ Butyrate	Maintains IFN-I signaling; regulates neonatal ILC2 activation through GPR41–IFNAR1 axis	Maternal antibiotic exposure reduces microbiota-derived butyrate, leading to downregulation of IFN-I signaling in neonatal ILC2s and causing elevated ILC2 numbers and heightened ILC2 activity in lungs. Results include epigenetic reprogramming of ILC2s, long-lasting susceptibility to allergic airway inflammation in adult offspring, and reversal of pathology by prenatal butyrate supplementation.	(57)
Neomycin and polymyxin B	*In vivo* (mice) + *in vitro* cell assays	8 weeks	0.6 mg/mL for neomycin and 120 units/mL for polymyxin B	uPLC	Enrichment of propionate-producing bacteria; reduction of other commensals; altered LPS-producing microbial components	↑ Propionate (increased propionate: acetate ratio)	SCFAs and LPS regulate lung immune responses; propionate dampens LPS-induced inflammatory signaling	Antibiotic-induced gut dysbiosis increased propionate: acetate ratio in colon lumen filtrates, which suppressed inflammatory responses. High propionate exposure in the lungs reduced immune containment of *S. aureus* pneumonia and reduced sterile lung inflammation.	(58)
Ampicillin, vancomycin, neomycin, metronidazole	*In vivo* (C57BL/6 male mice)	7 days	Ampicillin (1gm/lit), vancomycin (500mg/lit), neomycin (1gm/lit), 282 and metronidazole (1gm/lit) in the drinking water	-	Vancomycin: ↑ Verrucomicrobia, ↑ Proteobacteria; Neomycin: ↑ Bacteroidetes; AVNM: ↑ Proteobacteria with the lowest microbiome diversity	Decreased SCFAs in blood (antibiotic-dependent patterns)	SCFAs regulate host immunity and metabolism	Each antibiotic produced a distinct dysbiosis signature. Vancomycin markedly expanded Proteobacteria and Verrucomicrobia; neomycin increased Bacteroidetes; AVNM caused the most severe diversity loss. SCFA levels in blood decreased and correlated with altered cytokine responses (TNF-α, IL-10, IFN-γ).	(59)
Amoxicillin–clavulanic acid (AC)	*In vivo*, rhesus monkeys	14 days	6 mg/0.86 mg	GC-MS	Oral AC decreased acetate- and propionate-producing bacteria (specific phyla and genera reduced; e.g., Firmicutes/Bacteroidetes members)	Decreased acetic acid and propionic acid	SCFAs regulate BBB integrity and tight junction expression	Oral—but not IV—administration altered gut microbiota and caused a measurable increase in BBB permeability, especially in the thalamus. Reduced acetate and propionate correlated with increased cerebrospinal fluid/serum albumin ratio and Ktrans. Suggests gut microbiota–SCFA regulation of BBB in adult primates.	(60)
Ceftriaxone	Human neonates	7 days	80 µg/g	LC-MS	Antibiotic treatment induced gut dysbiosis, reducing overall microbial diversity and beneficial SCFA-producing bacteria	Decreased acetate, propionate, and butyrate in feces	SCFAs support the microbiota–gut–brain axis, neuronal maturation, microglial homeostasis, and myelination	Early-life antibiotics reduced SCFAs, impaired brain development, decreased IBA1 and MBP expression, and caused cognitive deficits. Microbiota restoration normalized SCFAs and improved neurological outcomes.	(61)
Ampicillin, neomycin, vancomycin, and metronidazole	*In vivo* (Conventional mice, germ-free mice)	7 days	Ampicillin (1 mg/mL), vancomycin (50 mg/kg), neomycin (100 mg/kg) and metronidazole (100 mg/kg)	GC-MS	–	Acetate, propionate, butyrate decreased	SCFAs act on vagal afferents to modulate gut–brain communication	Antibiotic perfusion decreased vagal activity in conventional mice; restored by intestinal filtrates from conventional but not germ-free mice. SCFAs activated vagal neurons and brainstem responses	(62)
Ampicillin, bacitracin, meropenem, neomycin, vancomycin	*In vivo* (Adult mice, intragastric treatment)	11 days	108.0 mg bacitracin, 108.0 mg neomycin, 43.2 mg ampicillin, 21.6 mgmeropenem, 6.48 mg vancomycin	NMR	Dysbiosis in Alphaproteobacteria; Caulobacterales;_Caulobacteraceae	SCFAs reduced	Modulate gut-brain signaling, neuronal function	Gut dysbiosis impaired novel object recognition, altered metabolite profile, disrupted serotonin transporter, neuropeptide Y, Brain-derived neurotrophic factor (BDNF), and GRIN2B expression in the brain	(63)
Vancomycin, enrofloxacin andneomycin	*In vivo* (Marmosets)	28 days	Vancomycin = 40 mg/kg, enrofloxacin = 10 mg/kg andneomycin = 20 mg/kg	GC-MS	decrease in alpha diversity	Decreased fecal SCFAs	Modulate gut-brain signaling via vagus nerve, GPCRs, enteroendocrine cells; reduce neuroinflammation and gut inflammation	Reduced SCFAs impaired gut-brain signaling; behavioral effects were less pronounced than bile acids or amino acids	(63)
Vancomycin	*In vivo* (Rat)	10 days	4 mg	GC	Fall in Enterobacteriaceae (~1.5 log), increase in Clostridium coccoides group and Bifidobacterium spp. (~2 log, not statistically significant)	Vancomycin reduced total SCFA concentration; a high-fiber diet altered SCFA profile (↑acetate, ↓propionate, ↓butyrate)	SCFAs regulate ion transport via FFA2/FFA3 signaling in the caecal epithelium	Vancomycin decreased total SCFAs and abolished propionate-induced anion secretion, whereas a high-fiber diet increased acetate but reduced propionate and butyrate; ion transport changes may also involve cholinergic signaling. Mast cell density increased after vancomycin; enterochromaffin cell density increased after a high-fiber diet. Highlights microbiota-epithelium interactions through SCFA/FFA signaling.	(64)
Broad-spectrum antibiotics (specifics not listed)	*In vivo* (Mice)	2 weeks	–	GC-MS	Antibiotic-induced gut dysbiosis disrupted corneal transcriptome and barrier function	Acetate, propionate, butyrate	SCFAs maintain corneal barrier integrity, regulate circadian gene expression, and support immune homeostasis	Antibiotic-induced gut dysbiosis disrupted corneal epithelial thickness, nerve density, sensitivity, and barrier integrity. SCFA supplementation restored corneal homeostasis, with SCFA receptors expressed in epithelial cells, macrophages, perivascular cells, and γδ T cells. This highlights the gut-eye axis and therapeutic potential of SCFAs in corneal dysfunction.	(65)
Antibiotic cocktail (vancomycin, metronidazole, ampicillin, neomycin)	*In vivo* (Mice with vascular calcification)	4 weeks	vancomycin (0.5 g/L), metronidazole (1 g/L), ampicillin (1 g/L), neomycin (1 g/L)	LCMS	Depletion of Bacteroidetes; general gut microbiota disruption	↓ Acetate (mainly), ↓ Propionate, ↓ Butyrate	SCFAs regulate vascular health, inhibit VSMC osteogenic transformation	ABX and vancomycin exacerbated vascular calcification in mice. Gut microbiota analysis showed reduced Bacteroidetes abundance and decreased serum SCFAs. Supplementation with acetate alleviated calcium deposition in the aorta and inhibited osteogenic transformation of VSMCs, possibly via glutathione metabolism and ubiquitin-mediated proteolysis. This highlights acetate as a potential therapeutic target in vascular calcification.	(8)
Ceftriaxone	*In vivo* (Wistar rat model)	14 days treatment + follow-up at days 1, 14, 56 post-withdrawal	300 mg/kg/day, intramuscular	Gas Chromatography (GC)	↑ Enterobacteria, ↑ E. coli, ↑ Clostridium, ↑ Staphylococcus spp., ↑ hemolytic bacteria; long-lasting dysbiosis	↓ Acetate, ↓ Propionate, ↓ Butyrate	Maintain epithelial barrier; regulate redox balance; FFA2/FFA3 receptor signaling; transporter (SMCT1, MCT1/4) function	Ceftriaxone treatment in Wistar rats caused long-lasting dysbiosis, reduced acetate, propionate, and butyrate levels, altered SCFA receptor and transporter expression, increased oxidative stress and hypoxia-inducible factor-1 (HIF-1)α/ERK1/2 activation, impaired epithelial barrier, and worsened colitis even 56 days after withdrawal.	(66)
Amoxicillin (AMX), Cefotaxime (CTX), Vancomycin (VAN), Metronidazole (MTZ)	*In vivo* (Wistar rats (n = 60, oral gavage))	10–11 days	Not stated in mg/kg (daily oral gavage as per study design)	GC	AMX, CTX, VAN → ↓ α-diversity; ↑ Proteobacteria. CTX → ↑ Bifidobacteriaceae. VAN → ↑ Lactobacillaceae, Verrucomicrobiaceae. MTZ → no microbial change.	AMX, CTX, and VAN significantly altered cecal SCFAs; succinate strongly correlated with Clostridiaceae 1 abundance and haptoglobin levels	SCFAs regulate permeability, inflammation, and epithelial integrity	AMX, CTX, and VAN treatments decreased microbiota diversity and altered SCFA levels, while MTZ had minimal effects; AMX and CTX increased plasma haptoglobin, CTX and VAN decreased intestinal permeability, and overall, antibiotic-induced microbial changes were linked to altered intestinal barrier function and inflammatory responses.	(67)
Lincomycin	*In vivo* (Infant mice (21-day old))	7 days (drinking water)	1 g/L or 5 g/L in drinking water	GC–MS	Significant reduction in microbiota diversity and major compositional shifts: decreased Enterococcus, Lactobacillus, and Muribaculaceae; altered phylum-level abundances; bloom of some genera at high dose.	↓ Acetate, ↓ Propionate, ↓ Butyrate, ↓ Valerate, ↓ Isobutyric acid, ↓ Isovaleric acid	SCFAs support colonocyte energy, maintain barrier integrity, and regulate immune signaling/TLR-mediated homeostasis	Impaired intestinal morphology (reduced villus height & crypt depth); altered expression of TLRs (TLR2, TLR3, TLR4), decreased inflammatory cytokines/TLR-NF-κB signaling; disrupted gut barrier/immune homeostasis in a dose-dependent manner.	(68)
Amoxicillin	*In vivo* (Wistar Han rats (male, n = 24))	7 days oral treatment + 7 days recovery	Not specified (oral gavage)	Untargeted metabolomics (caecal SCFA profiling)	↓ Alpha diversity; ↓ Lachnospiraceae and Ruminococcaceae (butyrate producers); ↑ Enterobacteriaceae; ↓ archaeal load	↓ Acetate, ↓ Propionate, ↓ Butyrate	SCFAs maintain the epithelial barrier, regulate immune signaling, and provide energy to colonocytes	Oral amoxicillin reduced SCFA levels and disrupted microbiota composition. The metabolome largely recovered 7 days post-treatment, but microbial diversity did not fully recover. There was no increase in luminal redox potential, and colonic gene expression related to barrier function and Reactive Oxygen Species (ROS) was largely unchanged, except that catalase was upregulated.	(69)
Gentamicin and/or Ceftriaxone	*In vivo* (Mice (recovery study post-antibiotics))	Not specified	–	¹H NMR-based metabonomics + DGGE microbiota fingerprinting	↓ Barnesiella, ↓ Prevotella, ↓ Alistipes; ↑ Bacteroides, ↑ Enterococcus, ↑ Erysipelotrichaceae incertae sedis, ↑ Mycoplasma	↓ Acetate, ↓ Propionate, ↓ Butyrate	SCFAs regulate epithelial barrier, immune signaling, and colonocyte energy	Antibiotic treatment reduced SCFA levels while increasing secondary bile acids and oligosaccharides, reflecting suppressed bacterial fermentation and protein degradation. The microbiota partially recovered over time, with correlations between fecal metabolites and bacterial taxa highlighting the functional role of specific bacteria in metabolite restoration.	(70)
Broad-spectrum antibiotics (ampicillin, vancomycin, metronidazole, neomycin, amphotericin B)	*In vivo* (Mice)	-(standard Antibiotic-induced microbiome depletion (AIMD) protocol)	-	GC-MS	↓ Firmicutes, ↓ Bacteroidetes; broad depletion of SCFA-producing bacteria	↓ Butyrate, ↓ Acetate, ↓ Propionate	SCFAs provide energy to colonocytes, regulate glucose homeostasis, and maintain gut signaling	AIMD caused depletion of SCFA-producing bacteria, leading to reduced luminal SCFAs and secondary bile acids. Colonocyte metabolism shifted toward glucose utilization, resulting in lower baseline serum glucose, improved insulin sensitivity, and altered cecal gene expression and Glucagon-like peptide-1 (GLP-1) signaling. Tissue remodeling in the colon reflected metabolic adaptation to SCFA deficiency.	(71)
Postnatal antibiotics (ampicillin, nafcillin, gentamicin, tobramycin, vancomycin)	Preterm infants (<36 weeks, <2000 g), stool and skin samples	1–3 weeks postnatal	–	Shotgun metagenomic sequencing, functional pathway analysis	↓ Butyrate-producing genera (Clostridium, Blautia) in antibiotic-exposed infants; antibiotic-naïve infants showed natural microbiome maturation	↓ Butyrate	Butyrate supports colonocyte energy, barrier integrity, and immune regulation	Early brief postnatal antibiotic exposure disrupted the microbiome developmental trajectory, preventing normal maturation of SCFA-producing bacteria and their metabolic pathways. This likely impairs SCFA production and contributes to adverse outcomes in preterm infants.	(72)
Vancomycin (Van) and Ciprofloxacin-Metronidazole (CiMe)	*In vivo* (Male and female mice)	2 weeks	Drinking water (vancomycin, 0.5 g/liter; ciprofloxacin, 0.2 g/liter;metronidazole, 1.0 g/liter)	NMR	↓ Firmicutes (females: Van & CiMe; males: Vanc), ↑ Proteobacteria (males: Van), and other structural changes are sex-dependent	↓ Acetate, ↓ Propionate, ↓ Butyrate	SCFAs regulate colonocyte energy, immune signaling, and gut homeostasis	Antibiotic exposure caused sex-dependent alterations in the gut microbiota and SCFA metabolism. Females experienced larger reductions in Firmicutes and SCFAs, along with decreased levels of alanine, branched-chain amino acids (BCAAs), and aromatic amino acids (AAAs) in colonic contents. Male mice were less affected, showing changes mainly after vancomycin. Microbe-metabolite correlations were stronger in females, highlighting sex-specific metabolic responses to antibiotics.	(9)
Low-dose erythromycin or azithromycin	Healthy adults	4 weeks	Twice-daily oral 400 mg erythromycin ethylsuccinate or twice-daily oral 125 mg azithromycin	NMR	Altered gut microbiome composition; reduced microbial carbohydrate metabolism and SCFA biosynthesis potential	↓ SCFA biosynthesis capacity (acetate, propionate, butyrate)	SCFAs support colonocyte energy, metabolic regulation, and carbohydrate metabolism	Four weeks of low-dose macrolide therapy altered gut microbiota composition and reduced SCFA biosynthesis capacity. These changes were associated with altered systemic metabolic biomarkers (serotonin, C-peptide). Murine transplantation confirmed that microbiome changes affected host metabolic homeostasis and gastrointestinal motility, while systemic immune regulation remained largely unaffected.	(73)
Trace-level Azithromycin (AZI) or Ciprofloxacin (CIP)	*In vivo* (Mice, drinking water exposure)	8 weeks	Drinking water at 100 and 200 ng/L, respectively	GC-MS	AZI: males showed changes in Firmicutes, Bacteroidetes, Proteobacteria, Fusobacteria, and Verrucomicrobia; Both AZI and CIP: abnormal microbiota maturation	↓ Acetate, ↓ Propionate, ↓ Butyrate (females affected)	SCFAs regulate colonocyte energy, gut signaling, and host metabolism	Trace antibiotic exposure disrupted gut microbiota maturation, altered SCFA production (especially in females), and shifted serum hormone and metabolome levels. AZI and CIP broadly disturbed host-microbe interactions, indicating that even low-dose environmental antibiotic exposure can perturb microbiota and metabolism in mice.	(74)
Van	*In vivo* (Female NMRI mice)	–	2 × 100 mg/kg/day	NMR	Firmicutes largely affected; overall strong shifts in gut microbial composition	↑ Fecal SCFAs (acetate, propionate, butyrate) observed in excretion	–	Van treatment strongly altered gut microbiota composition, particularly Firmicutes, leading to changes in fecal and urinary metabolites including SCFAs, uracil, and amino acids. Reduced urinary excretion of microbial co-metabolites (phenylacetylglycine, hippurate) was observed. Correlations between fecal SCFAs and urinary metabolites highlighted the close relationship between host metabolism and microbial metabolic activity.	(75)
Azithromycin (AZI) or Ciprofloxacin (CIP)	*In vivo* (Mice under a high-fat diet, drinking water exposure)	6 weeks	Drinking water atconcentrations of 100 ng/L and 200 ng/L, respectively	GC-MS	AZI altered microbial community structure; Lactobacillus was important in females; overall gut microbiota was significantly reshaped	↑ Acetate, ↑ Propionate, ↑ Butyrate (AZI in both sexes)	-	Trace antibiotic exposure under a high-fat diet induced significant shifts in gut microbiota and SCFA profiles. AZI increased SCFA production in both male and female mice. Both AZI and CIP altered serum hormone levels and metabolic profiles, increased male body weight, and disrupted microbe-SCFA and microbe-metabolite interactions, highlighting potential health risks in susceptible populations.	(76)
Metronidazole, Clindamycin, Erythromycin, Vancomycin	*In vitro* fecal fermentation; clinical in a child with propionic acidaemia	7-day oral courses	–	GC	Not specifically identified; gut propionate-producing bacteria suppressed	↓ Propionate	Propionate regulates metabolic load	Metronidazole caused the largest reduction in propionate production *in vitro* (77–84%) and *in vivo* (43% within 24 h, near complete elimination for 3 weeks). Other antibiotics also reduced SCFA production to a lesser extent. Plasma propionate concentrations decreased in parallel, indicating that intermittent courses of oral antibiotics can effectively suppress gut-derived propionate in children with propionic acidaemia.	(77)
Ciprofloxacin	Human volunteers	5 days	500 mg orally twice daily	GC-MS	Reduction of Bacteroides, Clostridium XIVa	↓ Acetate, ↓ Propionate	Energy supply; metabolic regulation	Ciprofloxacin caused temporary metabolic disruption and delayed recovery of gut microbiota by reducing key SCFA-producing bacteria such as Bacteroides and Clostridium XIVa, resulting in lower acetate and propionate levels.	(78)
Meropenem + Gentamicin + Vancomycin (last-resort cocktail)	Human study (12 healthy men)	4 days	Standard clinical doses: Meropenem IV (1 g q8h), Gentamicin IV (5–7 mg/kg/day), Vancomycin IV (15–20 mg/kg q8–12h)	GC-MS	↓ Bifidobacterium spp.; ↓ butyrate producers; ↑ blooms of Enterobacteriaceae, Enterococcus faecalis, Fusobacterium nucleatum	↓ Butyrate	Butyrate maintains epithelial integrity, anti-inflammatory activity, and colonocyte energy	Persistent loss of 9 common species (still absent at 6 months); selection for β-lactam, glycopeptide, and aminoglycoside resistance genes; altered recovery modulated by resistome; mild long-lasting imprint despite general resilience	
Wistar male rats	Oral amoxicillin	7 days	1 g/l	GC–MS	↓ α-diversity, ↓ archaeal load; ↓ butyrate-producing bacteria (Lachnospiraceae, Ruminococcaceae); ↑ Enterobacteriaceae; microbiome did not fully recover after 7 days	Significant ↓ in caecal SCFAs during treatment; full SCFA recovery by day 7 post-treatment	SCFA reduction reflects impaired epithelial barrier integrity and microbial metabolic activity	Amoxicillin disrupted gut microbiome & metabolome but did not increase luminal redox potential; minor changes in barrier-related genes; catalase gene ↑ post-treatment	(69)

↑, Increase; ↓, Decrease.

### 5.1 Antibiotic effect of SCFA on oxygen homeostasis and epithelial redox

The availability of oxygen is a critical factor in regulating reactive oxygen species, oxidative stress, and intestinal inflammation (79). Oxygen levels in the gut epithelium determine its choice between aerobic and anaerobic metabolism, which is strongly influenced by interactions between the host and its microbiota. High luminal oxygen can selectively favor the growth of aerobic pathogens and increase epithelial redox potential (79). The main reason for such an imbalance is antibiotic treatment, which decreases butyrate-producing bacteria. Reduced expression of peroxisome proliferator–activated receptor (PPAR) γ, a nuclear receptor that mediates the major oxidative function of butyrate, is the direct result of decreased butyrate availability within the gut lumen. Colonocytes consequently consume less oxygen, leading to increased availability of oxygen in the gut lumen and, thus, increased colonization by highly adhesive, aerobic Enterobacteriaceae. The outgrowth of Enterobacteriaceae correlates with enhanced epithelial redox potential. In contrast, partial recovery of the gut microbiota toward the pretreated state coincides with normalization of oxygen availability and epithelial redox balance. Together, these findings highlight the crucial role of a healthy microbiota in maintaining intestinal oxygen homeostasis as a first line of defense against pathogen overgrowth (79). A similar mechanism is elucidated in rodent studies: upon antibiotic treatment, the drop in butyrate lowers epithelial oxygen usage and enhances luminal oxygenation, conditions optimal for the proliferation of facultative anaerobic Proteobacteria (80). Limited butyrate availability shifts colonocyte metabolism toward anaerobic glycolysis, a pathway that does not consume oxygen, further contributing to elevated luminal oxygen (80). These effects have been described in mice treated with streptomycin, which selectively depletes butyrate-producing Clostridia (81).

Streptomycin treatment was shown to reduce the levels of SCFA and increase the redox potential in the cecum to values close to those obtained under aerobic conditions (82). In parallel, inducible nitric oxide synthase (iNOS), encoded by the NOS2 gene and usually repressed in human colonocytes through the SCFA-activated receptor PPAR-γ, is strongly induced in the cecum of streptomycin-treated mice (83, 84). Treatment with ceftriaxone for five days consequently induced expression of the redox-sensitive transcription factors Egr-1 and Sp-1 in the rat colonic mucosa (85). Furthermore, it was observed that Hypoxia-inducible factor 1-alpha (HIF1α) was raised twofold after the administration of ceftriaxone in the colonic mucosa. These findings suggest induction of proinflammatory changes associated with disruption of the oxidant–antioxidant balance, involving a direct connection between inflammation and hypoxia in the colonic mucosa (86).

### 5.2 Antibiotic-SCFA imbalance-induced metabolism

Antibiotic treatment reduces gut microbial diversity and alters community composition, leading to metabolic changes that affect not only the overall microbial ecosystem but also individual species (87). Recent studies have shown that low or sub-therapeutic doses of antibiotics can profoundly disrupt the composition and maturation of gut microbiota, leading to a range of adverse health outcomes that include weight gain and insulin resistance (88). In this regard, previous works have documented the impact of azithromycin and ciprofloxacin on gut microbiota development and the associated metabolic consequences in murine models at relevant concentrations (89, 90). Specifically, antibiotic exposure compromises the normal architecture and proper maturation of the gut microbiota by changing host metabolic pathways (76). An elevated Firmicutes/Bacteroidetes ratio has been linked with obesity and can be caused by dysregulated SCFA, specifically acetate, butyrate, and propionate, which are key modulators of energy homeostasis (76). Under high-fat diet conditions, azithromycin clearly augmented the production of SCFAs, accompanied by significantly enriched SCFA-producing bacteria such as Romboutsia and Lactobacillus, which may contribute to the rise in the synthesis of acetate, butyrate, and propionate, congruent with previous studies (91–93). Meanwhile, male mice exposed to trace levels of azithromycin and ciprofloxacin showed enhanced weight gain, while females did not have significant changes in body weight, indicating sex-specific susceptibility to antibiotic-induced metabolic disorder. In addition, Zhao et al. (76) demonstrated that the changes caused by azithromycin disrupted microbe–metabolite interactions rapidly, and the main contributors were Lachnospiracea_incertae_sedis in males and Bacteroides, Lactococcus, and Alistipes in females.

Consistent with the concept that intestinal ecology changes impact energy extraction efficiency, antibiotic treatment strongly decreased microbial fermentation products, such as SCFAs, in faeces, whereas SCFA precursors, including oligosaccharides, accumulated (94). Reflective of changes in bacterial composition, the relative proportions of butyrate and propionate to acetate were increased in antibiotic-treated animals. Host energy metabolism was also impacted, as reflected by increased urinary excretion of tricarboxylic acid (TCA) cycle intermediates, including citrate, 2-oxoglutarate, and fumarate, along with glucose, the ketone body D-3-hydroxybutyrate, and metabolites indirectly related to energy metabolism, such as alanine and lactate (94). In control animals, fecal SCFA levels, in particular propionate, showed a negative correlation with various urinary TCA cycle intermediates (citrate, 2-oxoglutarate, fumarate, malate, malonate), illustrating the intimate relationship between microbial SCFA production and host energy metabolism (94). Taken together, these data suggest that enhanced SCFA production by the gut microbiota, and thus increased SCFA bioavailability throughout the gastrointestinal tract, inhibits the TCA cycle through negative feedback, which is indicative of a metabolic shift that is influenced by microbial metabolites (94). Although these energy metabolic changes may emanate from either reduced bacterial activity or compositional changes, the findings emphasize the important association between gut microbiota and host metabolic control in antibiotic-treated animals.

Energy metabolism in the colon differs from that of other tissues in that colonocytes depend primarily on SCFAs, especially butyrate, for energy, whereas most other tissues use glucose predominantly (95, 96). In Holota et al. (66) study, immunoreactivity for monocarboxylate transporters (MCTs) was increased in the distal colonic mucosa after ceftriaxone treatment, indicating that, in the absence of luminal SCFAs, colonocytes tried to compensate by taking energy from the blood. These results add to the paradigm that intestinal bacteria are active regulators of host gene expression and specifically stimulate transcription of those host genes that are involved in the uptake and biological effects of bacterial fermentation products (66). Disruption in colonic microbiota metabolism was also related to the impaired activity of key antioxidant enzymes, superoxide dismutase (SOD) and catalase, along with a concomitant increase in the rate of lipid peroxidation as manifested by heightened levels of Malondialdehyde (MDA) in colonic mucosa (66). This proves that antibiotic administration can induce long-lasting oxidative disturbances within colonic tissue in rats.

By depriving the colon of its primary energy source, butyrate, enterocytes appear to compensate by increasing glucose utilization to meet their metabolic demands (71). This shift affects systemic glucose homeostasis by lowering serum glucose levels, enhancing insulin sensitivity, and increasing hepatic gluconeogenesis. Zarrinpar et al. (71) findings support previous evidence that antibiotic-induced microbiome depletion (AIMD) significantly influences glucose metabolism. For example, two weeks of norfloxacin-ampicillin treatment of both leptin-deficient (ob/ob) mice and Diet-Induced Obese (DIO) mice improved fasting blood glucose and oral glucose tolerance (97). Norfloxacin-ampicillin treatment of DIO mice also increased GLP-1 secretion and lowered serum LPS levels, which might help improve glucose homeostasis by enhancing gut barrier function and decreasing endotoxemia (98–100). Antibiotic administration in normal-chow-fed animals lowers blood glucose, enhances insulin sensitivity, and improves oral glucose tolerance, paralleling findings in Germ-free mice (101, 102). Furthermore, reduced luminal levels of SCFAs in Germ-free and antibiotic-treated mice alter the expression of Gcg (103).

Previous studies in Germ-free mice and Germ-free mice colonized with antibiotic-resistant bacteria demonstrated reduced active mitochondria and suppressed mitochondrial gene expression, culminating in epithelial cell death (104). Consistently, Zarrinpar et al. (71) observed reduced mitochondrial number and gene expression in AIMD mice, which likely underlie the elevated anaerobic glycolysis. Consequently, cecal enterocytes act as a “glucose sink,” increasing local glucose demand and perturbing systemic metabolic homeostasis. This effect appears to be specific to the colon, as the jejunum, which does not rely primarily on SCFAs for energy, exhibits reduced glycolysis under microbiome-depleted conditions (105). Additionally, exposure to antibiotics, including macrolides, significantly decreased the relative abundance of microbial pathways contributing to energy metabolism, including those involved in glycolysis, carbohydrate biosynthesis and degradation, the TCA cycle, and fermentation (73). Parallel experiments in mice showed that macrolide exposure altered the abundance of carbohydrate metabolism pathways, such as UDP-N-acetyl-D-glucosamine biosynthesis I and the Bifidobacterium shunt, and fermentation pathways that produce acetate and lactate from sugars. Analysis of fecal metabolites in mice verified these predicted functional changes by demonstrating a lower relative abundance of SCFAs (73). These findings are consistent with the effects of erythromycin on SCFA-producing taxa, such as members of the families Ruminococcaceae and Lachnospiraceae, which are capable of converting acetate and lactate into butyrate (24). Finally, in a study, Zeng et al. (8) found that glutathione depletion after broad-spectrum antibiotics or vancomycin-induced depletion of gut Bacteroidetes abolished the inhibitory effect of acetate on vascular smooth muscle cell (VSMC) osteogenic trans-differentiation, but MG132 had no such effect, suggesting that acetate may influence VSMC osteogenic transformation via glutathione metabolism. Previous work has shown that acetate alleviates oxidative stress by increasing levels of glutathione (GSH) and enhancing SOD activity (106).

### 5.3 Antibiotics and SCFA imbalance-mediated disruption of the physical barrier

SCFAs are essential for the viability and functional integrity of the large intestinal mucosa, including an intact epithelial barrier. In a study, Ballout et al. (64) noticed that vancomycin-induced decrease in SCFA may lead to the impairment of the barrier function of the cecal epithelium, whereas a high-fiber diet is expected to enhance epithelial integrity. Previous studies have also demonstrated a reduction in paracellular permeability in other segments of the large intestine, such as the colon, after vancomycin treatment (67, 107). This reduction in paracellular permeability can be ascribed to a modest increase in Bifidobacterium spp. after vancomycin administration, as these bacteria are known to preserve tight junction proteins, including claudin-4 and occludin, during colitis in mice (108).

Changes in the composition of gut microbiota induced by antibiotics can weaken the physical intestinal barrier by altering the expression of proteins in tight junction complexes and increasing IEC apoptosis (87). These effects promote the translocation of microbiota and pro-inflammatory mediators across the epithelium into the underlying lamina propria, where they initiate inflammation (87). For instance, Shi et al. (109) found that the treatment with ampicillin resulted in decreased expression of the tight junction complex proteins occludin and Zonula occludens-1 (ZO-1) in the colon. These changes were associated with an antibiotic-induced reduction in the levels of Lachnospiraceae and SCFAs (109). This may imply that antibiotic-induced alterations of symbiotic bacteria decrease epithelial exposure to commensals, leading to impaired intestinal barrier function and integrity (37). In Zhang et al. (68) study, exposure to lincomycin was associated with reduced villus height and crypt depth in the jejunum and ileum, respectively, indicating compromised integrity of the mucosa and more vulnerable gut environments. SCFAs are major substrates for intestinal epithelial cell energy metabolism and reportedly regulate the functional integrity of the epithelium (110, 111). Therefore, the low levels observed in the LM1 and LM5 groups may have promoted the reshaping of intestinal morphology (68).

Zhang et al. (68) also explored the impact of lincomycin on the expression of tight junction proteins such as occludin, claudin-1, and ZO-1. Li et al. (112) found that intestinal tight junction protein expressions increased with a decline in the Firmicutes/Bacteroidetes ratio in mice. Thus, changes in Firmicutes and Bacteroidetes by lincomycin in LM1 may be closely related to the intestinal tight junction barrier in this study (68). Higher levels of ZO-1 and occludin in LM1 suggest diminished intestinal permeability, which may restrain nutrient absorption temporarily and might account for the lower body weight gain in LM1 compared to CON and LM5 (68). Overall, the results indicate that reductions in both microbiota and SCFAs caused by antibiotics can alter both the morphology and barrier function of the intestine.

### 5.4 SCFA-immune modulation after antibiotic treatment

**Figure 2:**
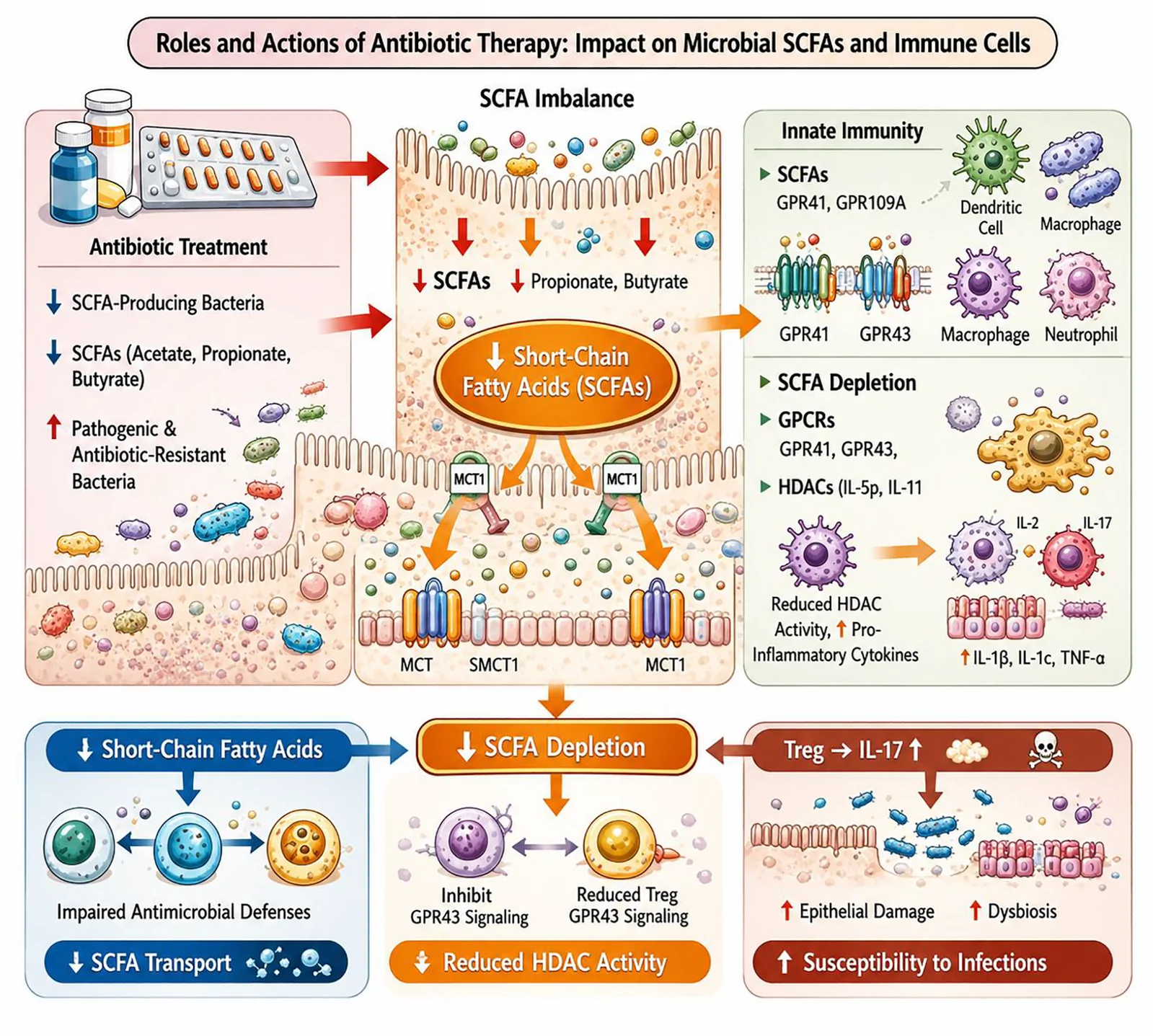
Antibiotic–induced SCFA imbalance and its consequences for immune regulation. Broad-spectrum antibiotic treatment (left panel) disrupts gut microbial diversity and depletes key SCFA-producing taxa (e.g., Clostridium clusters IV/XIVa, Lachnospiraceae, Ruminococcaceae, Bifidobacterium), while promoting the expansion of resistant and opportunistic bacteria. This dysbiosis results in reduced microbial fermentation of dietary fibers and an imbalance in SCFAs (central panel). Antibiotic exposure also alters epithelial SCFA handling by reducing luminal uptake via the sodium-coupled monocarboxylate transporter SMCT1 and modifying efflux through MCTs, accompanied by decreased expression of SCFA-sensing G protein–coupled receptors (GPR41/FFA3, GPR43/FFA2, and GPR109A) on intestinal epithelial and immune cells. SCFA depletion disrupts key signaling pathways that regulate immune homeostasis, including GPCR–Gi–cAMP signaling, HDAC inhibition, and microbial pattern-recognition receptor pathways (NOD1/NOD2 and TLR2/TLR4). As a consequence, innate immune cells such as macrophages, dendritic cells (DCs), and neutrophils exhibit heightened inflammatory responsiveness, increased NF-κB activity, and altered immunometabolism characterized by reduced oxidative phosphorylation and lipid metabolism. In parallel, adaptive immune balance is perturbed, with reduced Treg differentiation and IL-10 production, impaired Th17 development, and enhanced Th1-driven inflammation (right panels). Systemically and during critical developmental windows, antibiotic-induced SCFA depletion compromises epithelial barrier integrity, increases susceptibility to infection and inflammatory disease, and alters immune programming. Maternal antibiotic exposure reduces SCFA availability to offspring, dampening type I interferon (IFN) signaling and enhancing group 2 innate lymphoid cell (ILC2) activation, thereby predisposing neonates to allergic airway inflammation.

Since most immune cell subsets depend on SCFA-sensing pathways for their homeostasis, tolerance, and activation, SCFA depletion caused by antibiotics disrupts various immune processes (**Figure 2**) (55, 113). In this regard, antibiotics that deplete Gram-positive bacteria predominantly affect signaling through NOD2 and TLR-2, while Gram-negative targeting antibiotics signal through the NOD1 and TLR-4 pathways (114). In addition to being ligands for GPCRs, SCFAs are also inhibitors of histone deacetylases (HDACs). HDAC inhibition maintains anti-inflammatory responses by modulating dendritic cells (DCs) and macrophages, thereby positioning SCFA-induced HDAC inhibition as a key player in controlling innate immune responses and Nuclear factor kappa B (NF-κB) activity (115). SCFAs also regulate signaling through GPCRs, including GPR43, GPR41, and GPR109A, which are expressed by intestinal epithelial cells (IECs) and several immune cells (115).

In a study, Ballout et al. (64) found that SCFA-induced anion secretion was modified either by vancomycin treatment or by feeding a high-fiber diet. The inability to stimulate anion secretion by propionate after exposure to vancomycin could be due to altered Gi protein signaling, since both FFA2 and FFA3 receptors are coupled to Gi proteins (64). These inhibit the regulatory subunits of adenylate cyclases, thus lowering cytosolic cAMP levels. Because cAMP-dependent phosphorylation represents one critical step in activating the apical anion channel, the cystic fibrosis transmembrane conductance regulator (CFTR), interference at this level might explain the impaired SCFA-induced anion secretion (64).

The findings suggest that sodium-coupled monocarboxylate transporter 1 (SMCT1) mediates the uptake of luminal SCFAs, while MCTs are responsible for SCFA efflux into the circulation (66). The immunoreactivity of SMCT1 was significantly diminished after ceftriaxone exposure. Ceftriaxone treatment reduced colonic water absorption by about 30% (116). Holota et al. (66) also found FFA2 receptor expression on surface enterocytes and at the crypt base, and FFA3 receptors on surface enterocytes, goblet cells, and neurons within the myenteric ganglia of rat colonic tissue. Immediately following ceftriaxone exposure, levels of both FFA2 and FFA3 receptors were considerably decreased (66).

In a study, Xu et al. (57) demonstrated that offspring of mothers exposed to antibiotics showed an increased propensity towards airway inflammation, which was partly driven by augmented responses of group 2 innate lymphoid cells. Mechanistically, maternal antibiotic exposure diminished the production of SCFAs, which, through breastfeeding, dampened type I interferon signaling in neonatal ILC2s. The suppression of Interferon (IFN) 1 signaling promoted increased Group 2 innate lymphoid cell (ILC2) function, which resulted in elevated allergic airway inflammation in offspring (57). Antibiotic exposure during mid-pregnancy significantly increased both the number and activation of ILC2s in neonates, which translated into aggravated airway inflammation (57). Neonatal mice deficient in ILC2s were resistant to maternal antibiotic-induced type 2 inflammation, underscoring an important function for ILC2s in orchestrating pulmonary homeostasis at an early age. Microbiota analysis of the pups showed a consistent reduction in SCFA-producing species in offspring born to antibiotic-treated dams, but the overall microbial composition in the pups was different from that of the mothers, as expected (57).

Antibiotics directly suppress respiratory activity in immune cells, including those with phagocytic functions, impairing their overall immunological barrier capacity (117). In another way, antibiotics indirectly compromise immune defenses by altering the composition of gut microbiota. Changes in the microbiota induced by antibiotics can affect mucosal innate immunity, disrupt the balance between Th17 and Treg cells, and therefore influence intestinal immune responses (118). The intestinal immune homeostasis, maintained partly by SCFA-mediated pathways, is highly dependent on gut microbiota (87). Antibiotic treatment makes the macrophages hyperresponsive to bacterial stimuli, leading to enhanced production of inflammatory cytokines and heightened susceptibility to pathogenic infection. Even after recolonization by normal gut microbiota, antibiotic-treated mice showed increased Th1 cell-mediated colonic inflammation, indicative of a long-term, macrophage-dependent effect (87).

In vancomycin-treated mice, the marked reduction in colonic Treg cell populations was paralleled by diminished levels of Interleukin (IL)-10. This is very likely due to the loss of Clostridium species (clusters IV and XIVa), which are known to drive Treg cell differentiation and proliferation (119, 120). Exposure to vancomycin also eradicated members of the Cytophaga-Flavobacter-Bacteroidetes (CFB) phylum, associated with the differentiation of Th17 cells, from the gut microbiota (121). Similarly, low-dose penicillin suppressed the expression of Th17-associated genes and decreased the abundance of Lactobacillus and Allobaculum (121, 122). Abundances of these genera positively correlate with the expression of RORγt and IL-17, underscoring their role in the differentiation of Th17 cells (123). In summary, antibiotic treatment enhances intestinal inflammation by not only impairing T cell-mediated immunity but also aggravating susceptibility to infections (124).

Vancomycin-induced alteration of the gut microbiota also lowers SCFA production in the intestines and subsequently reduces GPR43 activation on Treg cells, thereby decreasing their colonic mucosal population (119, 125). Importantly, when mice are treated with vancomycin and SCFAs, colonic Treg cell populations remain unaltered (125). These observations confirm that antibiotic treatment-associated decreases in distal intestinal SCFA levels can decrease the population of Tregs in the colon. Antibiotic administration decreases SCFA levels, and butyrate production remains low during microbial recolonization (55). The role of SCFAs in our model was highlighted by the fact that butyrate inhibited intestinal macrophage hyperresponsiveness to microbial stimulation and abrogated deviant Th1 responses following recolonization (55). This is mediated at least in part by butyrate through the inhibition of HDAC activity, which represses inflammatory cytokine production in intestinal macrophages, although butyrate can act directly via its receptor GPR109a (126, 127). SCFAs also modulate the immunoregulatory function of other immune cells, including epithelial cells, DCs, neutrophils, and T cells (128).

Although Scott et al. (55) cannot completely exclude indirect effects of butyrate on cells other than intestinal macrophages, their data prove that butyrate directly decreases inflammatory cytokine production by intestinal macrophages. Finally, they uncovered a new mechanism where butyrate programs macrophage function by increasing oxidative phosphorylation (OXPHOS) and lipid metabolism, both of which are essential for alternative macrophage activation (55). Butyrate could impact the metabolic signature of intestinal macrophages directly as a metabolic substrate, through GPCR signaling, or via HDAC inhibition (20). In all, antibiotic exposure disrupts gut microbiota, depleting key SCFA-producing bacteria with long-lasting consequences for both mucosal and systemic immunity. Compromised availability of SCFAs impairs epithelial barrier integrity, alters macrophage metabolism and responsiveness, disturbs the balance between Treg and Th17, and increases susceptibility to inflammatory diseases and infections. These effects even reach into the next generation, as maternal use of antibiotics impairs SCFA-dependent regulatory pathways in neonates, particularly those regulating ILC2 activation and type 2 inflammation. Collectively, these findings position SCFAs as central mediators linking microbial homeostasis to immune regulation.

### 5.6 Antibiotics-microbiota SCFA axis, and neuroprotection

**Figure 3:**
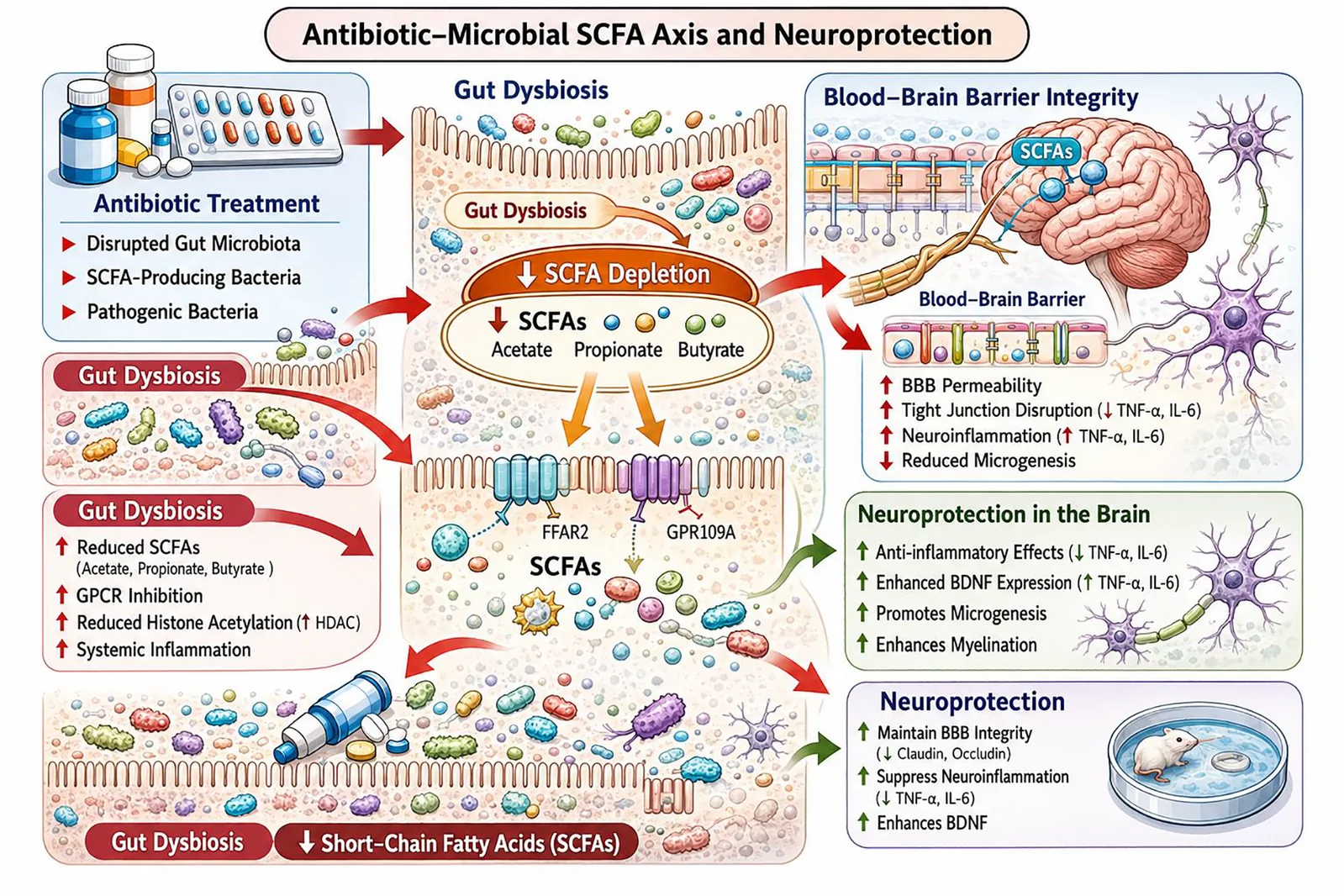
Antibiotic–microbial SCFA axis and neuroprotection. Antibiotic exposure (left panel) reduces microbial diversity and depletes key SCFA-producing taxa (e.g., Bacteroidetes and Clostridia), leading to decreased intestinal levels of acetate, propionate, and butyrate. This dysbiosis impairs microbial fermentation of dietary fiber and disrupts normal SCFA availability. In the gut (middle panel), reduced SCFA levels alter signaling through SCFA transporters (SMCTs and MCTs), G protein-coupled receptors (FFAR2/GPR43, FFAR3/GPR41, GPR109A, and TGR5), and epigenetic regulation via Histone deacetylase (HDAC) inhibition. SCFAs also indirectly modulate vagal afferent nerve activity through enteroendocrine and intermediary cells, thereby influencing gut–brain communication. Antibiotic-induced depletion of SCFAs weakens these signaling pathways and diminishes vagal tone. In the central nervous system (right panel), SCFAs contribute to blood–brain barrier (BBB) integrity by promoting tight junction protein expression (claudin and occludin) and limiting BBB permeability. Reduced SCFA availability following antibiotic treatment compromises BBB function, enhances neuroinflammation, and disrupts microglial maturation and myelination, as reflected by decreased IBA1 and myelin basic protein (MBP) expression. These changes are associated with impaired cognitive performance and altered neurodevelopment. The bottom panel highlights the heightened vulnerability during early life, where antibiotic exposure during critical developmental windows causes persistent SCFA deficiency, leading to long-term alterations in gut–brain signaling, neuroimmune homeostasis, and cognitive function.

Beyond their local effects in the colon and peripheral tissues, SCFAs are also considered important contributors to microbiota–gut–brain signaling (6). In addition to crossing the blood–brain barrier (BBB), SCFAs seem important for the maintenance of BBB integrity. The barrier’s selective permeability is crucial for controlling nutrient and molecular entry to the brain and thus for normal neurodevelopment and the maintenance of central nervous system (CNS) homeostasis (6). Consistent with such a role for SCFAs, germ-free mice show reduced expression of the tight junction proteins claudin and occludin, leading to increased permeability of the BBB from prenatal development into adulthood (129). While evidence exists for the presence of a “SCFA-brain axis” the exact mechanisms through which antibiotic exposure and microbial dysbiosis deplete SCFAs are poorly understood (**Figure 3**) (130, 131). According to rodent models, SCFAs contribute to this crosstalk by various means: modulating vagus nerve signaling, activating GPCRs in the gut and brain, influencing intracellular pathways, stimulating enteroendocrine cell receptors to regulate neuropeptide secretion, and diminishing both neuroinflammation and gut inflammation (132).

The findings revealed that, in accordance with the complex influence of the microbiota on vagal activity, Germ-free mice exhibited significantly reduced vagal afferent nerve activity compared with conventionally colonized specific pathogen-free (SPF) mice (62). This was restored by colonizing adult Germ-free mice with an SPF microbiota, whereas depleting gut bacteria in conventionally raised mice using oral antibiotics did not fully replicate this effect (62). Through the use of untargeted metabolomic profiling of small intestinal and cecal contents from conventionally colonized and microbiota-deficient mice, in combination with *in vivo* screening of selected microbial metabolites in the presence or absence of pharmacological antagonists, Jameson et al. (62) identified specific subclasses of microbiome-dependent molecules that activate vagal afferent neurons in a receptor-dependent manner when delivered to the small intestine. They further note a modest, transient rise in vagal afferent nerve activity that coincided with peak response during luminal perfusion of Transient Receptor Potential (TRP) agonists, suggesting that additional microbial metabolites may modulate vagal activity with smaller effect sizes (62). In addition, 30 small intestinal and 186 cecal metabolites, such as SCFAs, were increased in Germ-free and antibiotic mice versus SPF and conventional mice (62). These findings, combined with the published single-cell vagal transcriptomic data, suggest that microbiota SCFAs may indirectly activate the vagal afferent neurons, possibly through enteroendocrine cells or via other intermediary cells, by acting on FFAR2, TGR5, or TRPA1 receptors.

An emerging body of literature suggests that early-life antibiotic exposure disrupts gut microbiota-SCFA production and ultimately affects brain development and cognitive functions in neonatal mice. In a study by Zhang et al. (61), antibiotic-treated neonates showed reduced SCFAs in both humans and mice. In their mouse model, ceftriaxone caused profound dysbiosis, characterized by near-complete loss of Bacteroidetes and a dominance of Firmicutes. This disruption severely decreased SCFA levels—especially butyrate and propionate (61). Dysbiotic mice demonstrated impaired cognitive performance in the Morris water maze (MWM), including longer escape latency and reduced time in the target quadrant, suggesting learning and memory deficits (61). At the molecular level, antibiotic-induced dysbiosis did not significantly alter the neuronal marker neuron-specific enolase (NSE), indicating preserved neuronal differentiation. However, two critical components of neurodevelopment were impaired: myelination (reduced myelin basic protein (MBP) expression) and microglial function [reduced ionized calcium-binding adaptor molecule-1 (IBA1)] (61). Overall, the study highlights SCFAs as key mediators of gut–brain communication during early life and emphasizes the importance of cautious antibiotic use in infancy.

### 5.7 Antibiotics, SCFA, and anti-inflammatory response

The interplay between antibiotics and SCFAs represents a critical axis governing host immune homeostasis and inflammatory regulation. In Ray et al. (59) study, changes in the relative abundance of Firmicutes and Bacteroidetes due to antibiotic treatment differentially affected serum SCFA levels among groups. Increased Akkermansia and Lactobacillus genera in the cecum and colon after treatment with vancomycin correlated with higher colonic expression of the anti-inflammatory cytokine IL-10. Other studies also reported higher expression of gut anti-inflammatory cytokines with increased Akkermansia muciniphila abundance (133, 134). A pronounced expansion of the Bacteroides genus within the Bacteroidetes phylum after neomycin treatment correlated with higher expression of both IFN-γ and IL-10. In those mice, the decrease in Firmicutes along with the increase in Bacteroidetes was associated with lower serum butyrate and higher levels of acetate and propionate (59). Acetate specifically regulates host inflammatory responses through the promotion of IFN-γ expression by normalizing the IFN-γ promoter and histone acetylation in an acetyl-CoA synthetase (ACSS)-independent manner. These findings of increased IFN-γ and IL-10 expression may have thus been due to the neomycin-driven elevation of acetate (59). The AVNM (an antibiotic cocktail containing ampicillin, vancomycin, neomycin, and metronidazole)-treated group had a marked depletion in the abundances of major phyla, such as Firmicutes and Bacteroidetes, leading to a profound reduction in all three SCFAs-acetate, propionate, and butyrate-in serum compared with the controls and other antibiotic groups. This depletion of SCFAs was associated with higher colonic tumor necrosis factor-α (TNF-α) levels and lower expression of IL-10. On the contrary, the neomycin- and vancomycin-treated mice displayed increased levels of both acetate and propionate that were associated with greater production of IL-10 when compared to AVNM-treated mice, thereby emphasizing the role of specific SCFAs in modulating anti-inflammatory responses (59). In Zhang et al. (68) study, exposure to lincomycin resulted in the downregulation of TLR2 and TLR4, accompanied by lower levels of TNF-α, IL-1β, and P65 in mice. These observations agree with those made by Jena et al. (135), who documented lower ileal expression of IL-1β and IL-6 in mice treated with polymyxin B. Such findings could be related to a change in the abundance of Clostridium, which is known to regulate Treg activity and inflammatory responses (136). Antibiotic-induced decreases in microbial diversity could lower host stimulation by microbial-associated molecular patterns and, therefore, transiently decrease inflammatory responses. Furthermore, microbial metabolites are important regulators of cytokine production (137). Indeed, in Zhang et al. (68) study, low expression levels of cytokines, such as IL-1β, TNF-α, and IL-18, were related to significantly reduced levels of SCFAs, including acetate, propionate, butyrate, valerate, isobutyrate, and isovalerate. These observations suggest that the reduction of the microbiota due to antibiotic treatment may directly affect cytokine regulation.

## 6 Effects of antibiotic-associated SCFA imbalance in various diseases

As discussed above, the antibiotic-induced reduction of SCFA-producing bacteria affects the gut concentrations of acetate, propionate, and butyrate, influencing wide-ranging immunological and metabolic outcomes. Such antibiotic-associated depletions of SCFAs contribute to the development or exacerbation of numerous diseases, including infection, IBD, metabolic disorders, allergies, cancer, and neurological disorders (**Table 2**).

**Table 2:** Effects of antibiotic-associated SCFA imbalance in various diseases.

Disease	Study setting	Antibiotic type and dose	Exposure time	Affected bacterial dysbiosis	SCFAs	Effect	Reference
Type 2 allergic lung disease	*In vivo* (Mouse model)	Vancomycin (200 mg)	Early-life exposure	Significant depletion of SCFA-fermenting taxa in the gut microbiota	Decrease in SCFAs, especially butyrate	Loss of SCFA-producing bacteria leads to expansion and priming of lung ILC2s (↑ IL-2, IL-5, IL-13, IL-4), B1 cell activation, and ↑ innate IgE production → worsened allergic inflammation; dietary butyrate reverses the phenotype	(138)
Atopic dermatitis (AD)	*In vivo* (Ovalbumin (OVA)-induced mouse model)	Ampicillin (1 g/L), vancomycin (500 mg/L), ciprofloxacin(200 mg/L), imipenem (250 mg/L),and metronidazole (1 g/L)	2 weeks	Dysbiosis in early-life gut microbiota; specific taxa not detailed	SCFAs significantly decreased	Aggravated AD phenotypes: ↑ clinical score, ↑ transepidermal water loss, ↑ total IgE, ↑ IL-4 and IL-17, ↑ ILC3 in gut; ↓ FOXP3+ cells; probiotic or fecal supernatant restored SCFAs and ameliorated effects	(139)
AD	Human cohort study (CHILD Cohort Study)	Amoxicillin ampicillin azithromycin cefaclor cefalexin cefazolin cefdinir cefixime cefotaxime cefprozil ceftriaxone cephalexin clarithromycin clavulanic acid/or/potassium fidaxomicin metronidazole nitrofurantoin piperacillin ofloxacin sulfamethoxazole trimethoprim vancomycin	First year of life	↑ Tyzzerella nexilis, ↑ monosaccharide-utilizing bacteria; ↓ Bifidobacterium spp.; ↓ Eubacterium spp.; ↓ fermentative pathways	Reduced production of SCFAs due to loss of fermentative taxa	Early-life antibiotics disrupt fermentative microbiota → reduced SCFA production → dysregulated immune maturation → significantly increased AD risk in a dose-dependent manner (1 course aOR=1.67; ≥2 courses aOR=2.16). Microbiome alterations fully mediate the effect of antibiotics on AD development.	(140)
Allergic rhinitis (AR)	*In vivo* (Mouse model)	Vancomycin in drinking water	2 weeks before OVA sensitization	↓ Ruminococcus, ↓ Lactobacillus (SCFA producers); ↑ Escherichia-Shigella, ↑ Klebsiella	Fecal SCFAs significantly reduced	Dysbiosis increased total IgE, OVA-sIgE, IL-4, IL-5; enhanced nasal and colon inflammation; NaB supplementation restored SCFAs, reduced Th2 cytokines, increased IL-10/TGF-β1, and upregulated Foxp3	(141)
Food allergy	*In vivo* (Mouse model)	metronidazole (1 g/L), ampicillin (1 g/L), neomycin sulfate (1 g/L), and vancomycin(500 mg/L)	8 days	↓ Lachnospiraceae, ↓ Muribaculaceae, ↓ Ruminococcaceae (all SCFA producers); ↑ Enterococcaceae, ↑ Clostridiales	Significant decrease in total SCFAs and tryptophan metabolites; increased purine metabolism	Antibiotic-induced dysbiosis reduced SCFA-producing taxa, weakened intestinal tight junctions (↓ ZO-1, ↓ Claudin-3), increased intestinal permeability, elevated IgE/IgG1, and enhanced inflammatory responses; mice displayed more severe food-allergic reactions upon re-exposure	(142)
Primary sclerosing cholangitis(PSC)-associated Inflammatory bowel disease (IBD)	Single-arm interventional study in 15 patients with active colitis	Vancomycin, 125 mg	4 weeks	Reduced abundance of Lachnospiraceae, Blautia, Bacteroides; enriched Enterobacteriaceae, Veillonella, Akkermansia, Escherichia	Decreased SCFAs and secondary bile acids, including lithocholic acid derivatives	Oral vancomycin induced clinical remission in 12/15 patients, with reduced colitis activity (fecal calprotectin). Treatment led to gut microbiota compositional changes, downregulation of microbial SCFA metabolism and bile acid biotransformation pathways, and modulation of host genes involved in inflammation and extracellular matrix repair. Colitis relapsed after vancomycin withdrawal, indicating effects were reversible.	(143)
Colitis	*In vivo* (IL10-deficient mice)	Ampicillin, 1.0 g/L, novobiocin, at 1.0 g/L, metronidazole at 1.0 g/L and vancomycin hydrochloride	4 weeks	Dramatic reduction in gut microbiota abundance	Marked decrease in SCFAs in the colon	Antibiotic treatment exacerbated colitis, causing more severe intestinal inflammation and swollen cecum. Reduction in microbiota led to decreased Treg and Th1 cell proportions and lower SCFAs, disrupting intestinal homeostasis and worsening disease	(144)
Colitis	*In vivo* (mouse model)	Antibiotic cocktail (Abx): ampicillin (1 g/L), vancomycin (0.5 g/L), neomycin sulfate (1 g/L), metronidazole (1 g/L) administered in drinking water	2 weeks pretreatment before DSS	Reduced overall microbial diversity; increased inflammation-associated dysbiosis (Clostridium butyricum, Lachnospiraceae, Ruminococcaceae), increased pathogenic taxa.	Acetate, propionate, butyrate decreased	Abx-induced dysbiosis worsened inflammation and T-cell imbalance (↑Th1/Th17, ↓Th2). Probiotic treatment after Abx restored SCFAs, improved epithelial barrier, reduced inflammation, and shifted T-cell response toward protective Th2.	(145)
Hospital-acquired pneumonia (HAP)	Human patients	Imipenem (250 mg/L), metronidazole (1 g/L),vancomycin (500 mg/L), ciprofloxacin (200 mg/L) ampicillin (1 g/L)	6 weeks	Decreased gut microbiome diversity, depletion of SCFA-producing bacteria	SCFA levels decreased (general depletion)	Impaired pulmonary defense against MDR Klebsiella pneumoniae via compromised inflammatory monocyte (IM) FFAR2/3-dependent antibacterial activity	(146)
Salmonella enterica infection	*In vivo* (Murine model)	Streptomycin (20 mg)	12 hours	Loss of microbial population complexity in the ileum and cecum, especially Firmicutes	Acetate, propionate, and butyrate significantly decreased in the cecum	Streptomycin-induced SCFA depletion associated with increased Salmonella colonization, enhanced ileal inflammation, and worsened pathology	(147)
Vancomycin-resistant enterococci (VRE) intestinal colonisation	*In vivo* (Mice)	1000 µg/mL vancomycin, 25 µg/mL metronidazole, 152 µg/mL ceftriaxone, 139 µg/mL piperacillin/tazobactam, and383 µg/mL clindamycin	-	Gut microbiota disrupted; decreased microbial metabolites, including SCFAs	Individual SCFAs partially suppressed VRE growth; mixtures of SCFAs fully suppressed VRE	Antibiotic-induced SCFA depletion promotes VRE intestinal colonisation by reducing inhibitory metabolites and creating nutrient-enriched niches for VRE growth	(148)
Helicobacter pylori infection	Clinical study	Amoxicillin 1 g b.i.d. or t.i.d. with Vonoprazan 20 mg b.i.d.	7–10 days	Decreased abundance of Anaerostipes, Dialister, Lachnospira; altered microbial composition	Altered SCFA levels associated with decreased abundance of SCFA-producing bacteria in the H-VA group	Short-term VA therapy minimally affects gut microbiota and SCFAs; microbial composition is largely restored after treatment	(149)
Antibiotic-associated diarrhea (AAD)	Clinical case study	Amoxicillin-clavulanic acid (875 and 125 mg, respectively)	10 days	Marked loss of butyrate-producing bacteria; major restructuring of fecal microbiota diversity	Reduced butyrate availability	Large-scale gut microbiota disruption associated with diarrhea; resolution of symptoms occurred after antibiotic cessation with recovery of bacterial diversity and butyrate-producing taxa	(150)
Invasive fungal disease (IFD)	Clinical study+ *In vivo* (rat model)	Meropenem (2.4 mg/g) + vancomycin (0.9 mg/g)	14 days	Depletion of Clostridium species; reduction of Bacteroides; increased Enterococcus and pro-inflammatory conditional pathogens	Sharp decrease in SCFA production due to loss of Clostridium spp.	Antibiotic-induced microbial imbalance reduced SCFA-producing bacteria, impaired mucosal immunity (↓IgA, ↓IL-10), increased pro-inflammatory CD11c^+^ macrophages, and promoted susceptibility to Candida infection.	(151)
Candida albicans gastrointestinal colonization	*In vivo* (mouse model) and *in vitro* assays	Cefoperazone (0.5 mg/mL) in drinking water	7 days prior to C. albicans challenge	Marked depletion of gut bacterial community; antibiotic-sensitive taxa drastically reduced, enabling fungal overgrowth	Significant decline in acetate, butyrate, and propionate in cecal contents	SCFA depletion correlated with increased GI fungal burden. *In vitro*, physiological SCFAs inhibited growth, germ tube formation, hyphae development, and biofilm formation of C. albicans.	(7)
Clostridium difficile colonization susceptibility	*In vivo* (mouse model)	Tigecycline, ceftriaxone, piperacillin–tazobactam, linezolid; all administered subcutaneously for 3 days	Tested for colonization resistance at 1, 7, and 12 days after antibiotic completion	All antibiotics altered microbiota composition. Tigecycline caused the least microbiome disruption; ceftriaxone and linezolid caused moderate disruption; Piperacillin–tazobactam caused the most severe and persistent dysbiosis (up to day 7).	Antibiotics reduced SCFA-related metabolic pathways. Tigecycline caused minimal suppression of SCFA-associated metabolites; Others suppressed amino acids, bile acids, lipids, and SCFA-linked fermentation metabolites more strongly.	All antibiotics disrupted colonization resistance at 2 days; tigecycline, ceftriaxone, and linezolid recovered by day 7; piperacillin–tazobactam remained disrupted. Tigecycline’s milder metabolic impact correlates with faster restoration of colonization resistance.	(152)
Antibiotic-associated diarrhea (AAD)	Clinical study, 31 hospitalized patients; 9 received fecal microbiota enema	Clindamycin, penicillin, metronidazole, cefuroxime, netilmicin, amoxicillin, cephalothin, metronidazole, tetracycline	1–10 days	Marked global disruption of intestinal microflora (not species-resolved), significant loss of commensal bacterial functions	Significant reduction and disturbance of fecal SCFA patterns measured by GC	Fecal SCFAs were markedly reduced during AAD; fecal enema restored microbial function and SCFA profile, resulting in rapid clinical recovery within 4 days and no relapse for up to 18 months	(153)
Adipogenesis/obesity model	*In vivo* (C57BL/6 mice)	Florfenicol or azithromycin, 5 mg/kg/day	4 weeks	Altered gut microbiota composition, decreased richness and diversity; increased Firmicutes/Bacteroidetes ratio; declines in Christensenella, Gordonibacter, Anaerotruncus (florfenicol), Lactobacillus (azithromycin), and Alistipes, Desulfovibrio, Parasutterella, Rikenella (both)	Decreased SCFAs and secondary bile acids; increased primary bile acids	Antibiotic exposure increased adipogenesis and altered gut microbiota composition, SCFA production, and bile acid metabolism, suggesting a potential risk factor for obesity	(154)
Adiposity/metabolic alteration	*In vivo* (Mice)	Penicillin, vancomycin,penicillin plus vancomycin, chlortetracyclin	7 weeks	Shifts in taxonomic composition, such as the Lachnospiraceae bloom, an increase in the relative concentrations of Firmicutes compared toBacteroidetes	Increased colonic SCFAs; altered hepatic lipid and cholesterol metabolism	Early-life antibiotic exposure increased adiposity, altered gut microbiome composition, enhanced SCFA production, and disrupted metabolic homeostasis	(155)
Obesity	Obese prediabetic men	Amoxicillin or vancomycin	7 days	Decreased bacterial diversity; reduced Firmicutes involved in SCFA and bile acid metabolism	Altered plasma and fecal metabolite concentrations; SCFA-related pathways downregulated	Vancomycin altered gut microbiota composition and SCFA/bile acid metabolism but did not affect insulin sensitivity, adipocyte size, energy harvest, or systemic metabolic health at 8-week follow-up	(156)
Type 1 diabetes (T1D)	*In vivo* (NOD mouse model)	Macrolide (333 mg/L)	23 days	Loss of diversity; ↑ Enterococcus, Blautia, Enterobacteriaceae, Akkermansia; ↓ S24-7, Clostridiales, Oscillospira, Ruminococcus, Anaeroplasma	Cecal butyrate decreased	Accelerated T1D onset in males; dysregulated ileal gene expression, altered histone modifications, impaired Treg development, epigenetic and metabolic disruption; long-term immune dysregulation	(157)
T1D	*In vivo* (Rat model: advanced-stage T1D (AST1D) and age-matched controls (AMCs)	Vancomycin (Vanc); Ciprofloxacin + Metronidazole (CiMe) (10 mg)	2 weeks	Altered gut microbiota; stronger shifts in AMC rats than AST1D rats	SCFA metabolism altered	Antibiotic-induced gut dysbiosis caused metabolic disturbances, including energy, amino acid, and SCFA metabolism; Vanc had a stronger impact than CiMe; effects differ between diabetic and control rats	(158)
Neointimal hyperplasia/arterial injury	*In vivo* (Rat)	Vancomycin	4 weeks	Altered gut microbial composition	Butyrate decreased by 69%	Ratstreated with oral vancomycin had exacerbated neointimal hyperplasia development after carotid angioplasty.	(159)
Developmental hypertension	*In vivo* (Rat)	Minocycline, 50 mg/kg/day orally	12 weeks	↑ F/B ratio, ↓ Lactobacillus, ↓ Ruminococcus, ↓ Odoribacter	↓ Plasma acetic acid and butyric acid; altered SCFA receptor expression	Maternal exposure to minocycline during pregnancy and lactation led to elevated blood pressure in adult male offspring, associated with decreased SCFA levels, altered gut microbiota composition, and aberrant activation of the renin–angiotensin system.	(160)
Hematopoietic stem cell transplant (HSCT) complications/graft-versus-host disease(GVHD)	Children undergoing allogeneic HSCT	Piperacillin or tazobactam, second-line meropenem, aminoglycosides, metronidazole, and clindamycin	During and after HSCT (first 14 days)	Loss of commensal SCFA-producing bacteria, overall reduced microbiome diversity	↓ Butyrate and propionate levels	Antibiotic exposure after HSCT significantly reduced SCFA-producing commensals, lowering butyrate and propionate levels, which was associated with increased risk of GVHD and may exacerbate gut injury in pediatric HSCT patients.	(161)
Intestinal GVHD after allo-HSCT	*In vivo* (Mouse model of allo-HSCT)	Gentamicin (dose not specified)	28 days	Altered gut microbiota; decreased Clostridium levels	Butyrate	Gentamicin reduced GVHD severity and mortality by modulating the gut microbiota. However, sodium butyrate supplementation after gentamicin treatment worsened GVHD, reducing colon length, thinning the mucus layer, inducing goblet cell apoptosis, inhibiting Muc2 expression, and impairing intestinal stem cell proliferation. Timing of butyrate exposure critically determined its effect on GVHD outcome.	(162)
Breast cancer	*In vivo* (Mouse model)	1mg/ml Amphotericin B, 25mg/ml Vancomycin, 50mg/ml Neomycin,50mg/ml Metronidazole, 1mg/mlAmpicillin and or 44mg/ml Cephalexin	5 days	Depletion of butyrate-producing genera: Odoribacter, Anaerotruncus, and increased Bacteroides	Butyrate decreased	Accelerated tumor growth; metabolic dysregulation (protein and lipid metabolism); intratumoral immunity unchanged	(163)
Hematological malignancy	Clinical + *in vivo* (rat model)	Cephalosporin, Quinolones, Glycopeptides, Carbapenems,	**-**	↓ Lactobacillus, ↑ Enterorhabdus. Rats: – Control: ↑ Lactobacillus – AAD: ↑ Enterorhabdus – AAD + Low-SCFA: ↑ Turicibacter – AAD + High-SCFA: ↑ Clostridium IV	Reduced fecal SCFAs.	SCFAs alleviate intestinal inflammation, reduce colon shortening, restore epithelial barrier integrity (↑ CLDN3 & ZO-1, ↓ PLVAP), reduce pathology, and beneficially shift gut microbiota composition.	(164)
Traumatic Brain Injury (TBI)	*In vivo* (Mouse model)	Ampicillin, gentamicin, metronidazole, vancomycin	2 days	Dramatic decrease in microbiota diversity and abundance; major shifts after ABX and second TBI	SCFA production	Broad-spectrum ABX diminished existing microbiota and reduced cortical damage, apoptotic cell density, and microglial/macrophage activation after second TBI—despite impairing gut integrity.	(165)
Depression	Human observational study (community-dwelling older adults)	Sulfadiazine, azithromycin, tetracyclines, veterinary antibiotics (VAs); levels detected via urinary biomarkers (dose not specified)	Chronic exposure, duration not specified	Not directly assessed	↓ Caproic acid, ↓ iso-butyric acid, ↓ iso-caproic acid (iso-CA) (especially with tetracycline); ↑ β-hydroxybutyric acid	Antibiotic exposure associated with higher depressive symptoms (↑ GDS-30 scores). Reduced iso-CA levels inversely correlated with depression severity. Iso-CA partially mediated depression risk associated with ofloxacin (25.3%) and tetracycline (46.3%), suggesting antibiotic-driven SCFA imbalance contributes to depression.	(166)
Postoperative cognitive dysfunction (POCD)	*In vivo* (Mouse model)	Cefazolin (dose not specified; perioperative administration)	14 days	Dysbiosis was reversed for several fecal bacteria, including β-, γ/δ-, and α-Proteobacteria; Bacteroidetes normalized	POCD reduces nearly all SCFAs; cefazolin restores ↑ acetic acid and ↑ propionic acid	Cefazolin improved cognitive impairment by preserving blood–brain barrier integrity and upregulating tight-junction proteins (ZO-1 and OCLN). It reversed gut dysbiosis induced by anesthesia and surgery and restored the levels of key SCFAs, particularly acetate and propionate. These combined effects contributed to the attenuation of POCD.	(167)
CUMS-induced depression-like behaviors	*In vivo* (Adolescent rat model exposed to chronic unpredictable mild stress)	Rifaximin, 150 mg/kg, intragastric	4 weeks	Increased abundance of Ruminococcaceae and Lachnospiraceae	Increased brain butyrate	Rifaximin reduced depressive-like behavior by reshaping gut microbiota composition and enhancing butyrate production. It promoted anti-inflammatory microglial function and prevented CUMS-induced defects in hippocampal neurogenesis. These microbiota–SCFA–microglia interactions contributed to antidepressant and neuroprotective effects.	(168)

↑, Increase; ↓, Decrease.

### 6.1 Antibiotic-associated infections

Antibiotic-induced changes in the intestinal microbiota can decrease carbohydrate absorption by inhibiting SCFA-mediated fermentation (169). The resulting accumulation of unfermented carbohydrates in the colon increases luminal osmolarity, drawing water into the intestinal lumen and causing osmotic diarrhea (170). *In vitro* experiments have shown that normal individuals have a depressed capacity to metabolize carbohydrates in the presence of antibiotics (171). Consistently, low levels of SCFA have been recorded both in the presence of antibiotics that are active against anaerobic bacteria in healthy subjects and in patients receiving such antibiotics (172).

Prolonged antibiotic exposure is a known risk factor for healthcare-associated Clostridioides difficile infection (173). Antibiotic-mediated disturbances in gut microbiota create an environment that promotes C. difficile spore germination and outgrowth (174). As bacterial metabolic end products, SCFAs, which may inhibit C. difficile infection (CDI), are diminished due to antibiotic interference with microbial metabolism. Administration of clindamycin and ampicillin to healthy individuals reduced SCFA concentrations, although metronidazole had a minimal impact (175). Cefoperazone treatment also induced significant changes in mouse gut microbial metabolism, most notably a significant decrease in valerate, which predisposed mice to CDI infection (176). Mcdonald et al. (177) replicated this finding using a CDI chemostat model, where the addition of clindamycin reduced valerate levels and increased total viable counts of C. difficile. Antibiotics also transiently increased succinate levels in the mouse intestines, a metabolite known to support the growth of C. difficile (178). These data clearly show that while antibiotics can suppress C. difficile directly, they concomitantly reduce SCFA-based defenses. Hence, this makes careful consideration of antibiotic therapy an important aspect of managing CDI.

The findings suggest that streptomycin-induced changes in SCFA distribution and abundance within the gut may be highly relevant for Salmonella virulence (147). Pre-exposure to streptomycin before infection with Salmonella resulted in extreme intestinal pathology, including severe cecal inflammation and, as described here, exacerbated ileal pathology (179). However, the ileal changes could not be directly related to altered SCFA levels at this site because the SCFA profile of untreated mice was already supportive of invasion, and it was unaltered by streptomycin treatment (147). Conversely, the cecal contents of streptomycin-treated mice were characterized by reduced SCFA levels, especially butyrate. These findings thus indicate that streptomycin reduces butyrate’s inhibitory effect on bacterial invasion in the cecum, thereby promoting Salmonella invasion and its associated cecal pathology in this model.

Dörner et al. (146) showed that the depletion of SCFA-producing bacteria following antibiotic treatment impairs the microbicidal activity of inflammatory monocytes, thus weakening antibacterial immune defenses in the lung. Antibiotics not only target pathogenic organisms but also the resident microbiota, which compromises colonization resistance and allows intestinal overgrowth of opportunistic pathogens like K. pneumoniae (180–182). Accordingly, Dörner et al. (146) data suggest that treatment with commonly applied parenteral broad-spectrum antibiotics-most of the patients received either carbapenems or piperacillin-tazobactam-further compromises pulmonary antimicrobial immunity by depleting SCFA producers and functionally impairing inflammatory monocytes. Although Dörner et al. (146) understanding of how different antibiotic classes impact the microbiome remains incomplete, the results are consistent with recent reports that demonstrate both piperacillin-tazobactam and carbapenems severely disrupt gut microbiota and deplete anaerobic commensals (183, 184). SCFAs have been shown to modulate the production of reactive oxygen species, phagocytosis, and chemotaxis of polymorphonuclear cells through FFAR2, promote macrophage differentiation through FFAR3, and imprint antimicrobial programs in macrophages through HDAC3 inhibition (146). Dörner et al. (146) extend these findings by showing that SCFAs augment monocyte-microbicidal function via FFAR2/FFAR3 activation to enhance phagocytosis, endolysosomal acidification, and lysozyme function critical for the control of bacterial lung infection *in vivo*. The impaired microbicidal function of inflammatory monocytes in mice colonized with antibiotic-altered microbiota, or in FFAR2/FFAR3-deficient animals, was consistent with reduced expression of genes encoding key regulators of phagocytic function, lysosomal degradation, and lysozyme activity.

Evidence suggests that perturbations in immune regulation are associated with invasive Candida albicans infections, and antibiotic-induced gut dysbiosis is a profound risk factor for increased C. albicans colonization and dissemination in immunocompromised patients and in individuals with AAD (185, 186). Treatment with broad-spectrum antibiotics enhances host susceptibility to C. albicans colonization in the gastrointestinal tract, which is often the source of systemic infection (185, 187). In Guinan et al. (7) study, the cefoperazone-treated mice were more susceptible to C. albicans infection and had significantly lower levels of SCFAs in cecal contents as compared to the control groups. They further found that changes in SCFA levels induced by antibiotics directly influence C. albicans growth and morphogenesis and thus enhance its colonization in the gut of cefoperazone-treated mice (7). The virulence of C. albicans is directly linked to its ability to undergo a morphological transition from harmless yeast cells to virulent hyphal cells; thus, limiting this morphological transition severely decreases its virulence (188, 189). *In vitro* experiments showed that the average SCFA concentrations in control mice (acetate: 25 mM, butyrate: 12.5 mM, propionate: 12.5 mM) significantly inhibited the growth of C. albicans, while the reduced concentrations of SCFAs found in the antibiotic-treated mice (acetate: 12.5 mM, butyrate: 3.25 mM, propionate: 3.25 mM) had only minor inhibitory effects (7). These findings collectively suggest that antibiotic-mediated reductions in cecal SCFAs promote the growth and gut colonization by C. albicans.

Huang et al. (151) also confirmed that SCFA-producing Clostridium species were nearly eliminated from the intestinal microbiome in a rat model of invasive fungal disease (IFD), while Enterococcus increased significantly following carbapenem therapy. Correspondingly, the expression of IgA and IL-10 in the intestinal tissue of fungus-infected rats decreased markedly after antibiotic treatment, suggesting that antibiotics can disrupt intestinal immune homeostasis by altering neonatal gut microecology (151). Huang et al. (151) results indicated that the abundance of SCFA-producing Clostridium species and Bacteroidales, including acetate- and butyrate-producing bacteria, was drastically lower in both IFD infants and rats. The Clostridium clusters XIVa and Bacteroides have been reported to induce HIF-1α and IL-37 and suppress C. albicans colonization (190). Animal studies demonstrated that Clostridium clusters IV, XIVa, and XVIII are involved in SCFA production, which induces colonic Tregs (119, 191). Huang et al. (151) found severe loss of C. butyricum within the intestinal microbiota of IFD rats. KEGG metabolic pathways related to SCFA metabolism were linked to shifts in the abundance of these SCFA-producing probiotics. They suggest that depletion of Clostridium sp. rendered the rats more susceptible to fungal colonization. Taken together, antibiotic treatment resulted in decreased levels of Clostridium sp. and Bacteroides sp. with increased Enterococcus sp., which was inversely related to lower expression of IgA and the anti-inflammatory cytokine IL-10, factors considered important for intestinal homeostasis (151). It therefore appears that impaired SCFA-mediated intestinal immune resistance supports invasive fungal colonization.

### 6.2 Autoimmune diseases

Recent evidence has suggested that early-life gut microbiome perturbations can affect the development of the immune system and hasten the onset of the autoimmune disease type 1 diabetes (T1D), characterized by the destruction of pancreatic insulin-producing cells (157). Zhang et al. (157) aimed to determine whether a single early-life pulse of antibiotics, administered during postnatal days P5–P10, was sufficient to promote the development of T1D in nonobese diabetic (NOD) mice. Indeed, Zhang et al. (157) found that an even brief early-life course of antibiotics induces extensive changes in microbiota, acting through microbial metabolites and host cellular interaction to effect cascades of downstream host-mediated events. These events reprogram innate and adaptive immunity to accelerate and enhance the onset of T1D. Zhang et al. (157) analyses indicate that antibiotic-induced remodeling of the gut microbiome reduced intestinal SCFA levels, disrupted histone Post-translational modifications (PTMs), and repressed mucin gene expression—factors that together impair the development of innate intestinal immunity. Indeed, butyrate and propionate have been shown to enhance histone H3 and H4 acetylation and H3 methylation, modulate the Mucin 2 (Muc2) promoter, and increase Muc2 mRNA levels (192). The reduction of SCFAs in this study is consistent with the parallel decrease in Muc2 expression.

Jia et al. (193) revealed that butyrate reduced vancomycin-exacerbated inflammation and decreased the recruitment of IFN-γ^+^ T cells in the pancreas of female offspring. This work focused on butyrates’ *in vivo* effects on various T-cell subsets. Expansion of CD4^+^ or CD8^+^ effector T cells that produce proinflammatory cytokines such as IFN-γ, or diminishment of Treg number or function, can induce β-cell destruction and promote insulitis to contribute to T1D development (193). IFN-γ also promotes T1D disease progression by enhancing the homing of diabetogenic T cells to the pancreas, an essential step for insulitis. Tregs in inflamed islets primarily act on CD8^+^ T cells to reduce their recruitment and to suppress both IFN-γ production and signaling (193). Collectively, these data suggest that pancreatic immune tolerance to prevent or treat T1D needs to be achieved simultaneously in a manner that modulates IFN-γ^+^ T cells (Tc1 and Th1 cells) and Tregs. In Jia et al. (193) study, cytokine analysis showed that butyrate treatment significantly suppressed pancreatic IFN-γ secretion in maternal NOD mice and their female offspring. Butyrate not only suppressed IFN-γ^+^ T cells but also enhanced the population of pancreatic Tregs. These findings indicate that the protective effect of butyrate against T1D might be attributed to its dual action: enhancing Treg populations and limiting IFN-γ^+^ T-cell recruitment in the pancreas (193). Importantly, butyrate administration during pregnancy and lactation significantly prevented vancomycin-accelerated T1D development in female offspring, pointing to early life as a critical window of butyrate-mediated protection and vancomycin-induced disease susceptibility (193). During this perinatal period, the intestinal environment is experiencing major developmental changes, and disturbances at this stage may have long-lasting effects on pancreatic immune homeostasis. These findings support the notion of a perinatal window during which maternal gut perturbations shape neonatal innate and adaptive immunity and, by extension, impact T1D susceptibility in the offspring.

Studies have shown that early-life antibiotic exposure can disrupt gut microbiota, leading to decreased microbial diversity and distinct compositional changes that play a critical role in the development of IBD (194, 195). At the phylum level, antibiotic treatment during early life decreased Bacteroidetes while increasing Proteobacteria, Firmicutes, and Verrucomicrobia, with these alterations persisting even after dextran sodium sulfate administration (195). At the family level, early-life antibiotic exposure resulted in a higher abundance of proinflammatory Enterobacteriaceae and a reduced abundance of commensal bacteria, such as the S24–7 family and Prevotellaceae (194). The results showed that antibiotic pretreatment could ameliorate intestinal inflammation, as evidenced by improvement in body weight, prevention of colon shortening, and reduction of histological injury, compared with dextran sulfate sodium (DSS)-induced colitis mice (145). Antibiotic pretreatment seemed to regulate T cell immune responses, especially affecting CD4+ T cells, which may indicate an immunomodulatory effect in colitis (145). The relative abundance of Firmicutes was lower in mice with DSS-induced colitis, but increased after Cognitive bias modification (CBM) intervention (145). Previous studies indicated that Proteobacteria dominated the intestine of mice pretreated with an antibiotic alone (196). CBM intervention reduced the abundance of potential pathogenic bacteria, including Proteobacteria, Fusobacteria, and Enterobacteriaceae, while increasing the abundance of SCFA-producing taxa. Notably, the abundance of butyrate-producing bacteria is typically low in IBD patients compared to that in healthy controls (197). Accompanying such microbial changes, the total concentration of SCFAs in feces increased after CBM intervention, with a similar increasing trend for each individual SCFA (acetate, propionate, isobutyric acid, and butyrate) (145). These findings indicate that antibiotic pretreatment creates a favorable environment for the proliferation of beneficial bacteria and that CBM can more effectively restore dysbiosis and metabolic imbalance in the gut. Moreover, most gut bacteria being depleted by antibiotic treatment likely allowed CBM to rapidly colonize the intestinal tract, thus enhancing its anti-inflammatory effects. Although SCFAs, known to regulate colonic Treg homeostasis, were reduced, vancomycin’s therapeutic benefits in IBD were thought to occur independent of SCFA-mediated pathways (198). These findings occurred in parallel with a recent mouse model of the closely related disease PSC, where vancomycin was shown to exacerbate biliary inflammation, reduce SCFA production, and expand pathogenic subsets of Escherichia and Enterococcus (199). Microbial and metabolic changes aside, clinical and biochemical improvements were noted, further suggesting that the beneficial effects of OV in PSC-IBD occur independently of SCFA production or Proteobacteria abundance.

### 6.3 Graft-versus-host disease

Hematopoietic stem-cell transplantation (HSCT) predominantly damages the gastrointestinal system, and severe intestinal Graft-versus-host disease (GVHD) remains a major factor in poor prognosis among post-HSCT patients (200). Antibiotics are widely used to treat various infections after allo-HSCT, and recent research has shown that gut-decontaminating prophylactic antibiotics can alter the severity of intestinal GVHD (201). Although some studies have indicated that the inhibition of Clostridium proliferation could support intestinal homeostasis or be used to treat intestinal diseases, the specific role of Clostridium in GVHD remains to be determined (162). There is also an increasing number of reports suggesting that SCFAs, especially butyrate, are essential in maintaining intestinal metabolic homeostasis. Although several reports have demonstrated the protective effects of butyrate on GVHD, Golob et al. (202) emphasized that butyrate has a double-edged effect depending on the timing of exposure. However, broad-spectrum antibiotics are still critical to treat infections in allo-HSCT patients, although some antibiotics are associated with increased intestinal GVHD and lower overall survival (162). To study the effect of gentamicin treatment on GVHD, feces from mice in the different groups were submitted for 16S rRNA sequencing. Clostridium was significantly decreased in the feces of mice treated with gentamicin. Notably, butyrate is an important metabolite of Clostridium in the intestine. In prior reports, sodium butyrate was identified as an HDAC inhibitor that significantly prolongs survival in GVHD mice (203, 204). In contrast to these reports, Zhang et al. (162) results showed that sodium butyrate administration reduced the survival rate of GVHD mice and aggravated gastrointestinal mucosal injury. On the contrary, pre-transplantation administration of gentamicin significantly extended the survival of GVHD mice and alleviated the severity of the disease. Fecal sequencing showed a significant decline in butyrate-producing bacteria in the gentamicin-exposed group. Of particular note is that supplementation of sodium butyrate post-transplantation abrogated the protection afforded by gentamicin (162). Collectively, these findings provide new insights into the complex interrelations between antibiotics, SCFAs, and GVHD pathogenesis, suggesting that the timing and context of SCFA exposure are critical in dictating disease outcomes.

Children undergoing HSCT were directly studied in the present work to explore a potential mechanism by which microbiota alterations are linked to outcomes after transplant through direct reductions of luminal SCFAs (161). Romick-Rosendale et al. (161) focused on the early transplant period, when clinical gut injury is most pronounced, as bacterial translocation and danger-associated molecular patterns during this time are believed to initiate acute GVHD in the gut, skin, and liver, significantly contributing to post-HSCT mortality. They found a gradual decrease in SCFAs within the intestinal lumen during the initial weeks after HSCT, with the most significant decreases for butyrate and propionate (161). Romick-Rosendale et al. (161) investigated the expression of most butyrate transporters and receptors in mucosal biopsies obtained from children with GVHD and found that the majority were unaltered, with some transporters upregulated compared to earlier mouse studies. There was found a loss of Firmicutes and other anaerobic taxa, including Ruminococcus, which correlated well with the reduced levels of SCFAs. The higher the exposure to antibiotics active against anaerobic bacteria, the lower the butyrate and propionate levels, suggesting the need to avoid unnecessary anti-anaerobic antibiotic use post-HSCT (161).

Mathewson et al. (205) suggested decreased expression of a single butyrate transporter and receptor as a possible mechanism of reduced colonic butyrate in mice with GVHD. In contrast, the present study investigated eight transporters and three receptors and demonstrated no consistent alterations in the specific transporter and receptor reported by Mathewson; instead, expression changes across other transporters and receptors were variable. Thus, the clinical relevance of butyrate transporter changes in the human colon during GVHD remains uncertain. In all, these findings detail a possible mechanism through which microbiota perturbation and SCFA depletion increase the risk of gut GVHD and post-HSCT mortality. Given the overall low incidence of GVHD in our cohort—likely a consequence of participant young age—these findings should be validated in larger data sets (161). These data reinforce the notion of interventions that maintain microbiota diversity, possibly through enteral supplementation, while emphasizing the importance of judicious antibiotic use post-HSCT to preserve anaerobic microbial populations.

### 6.4 Allergic diseases

**Figure 4:**
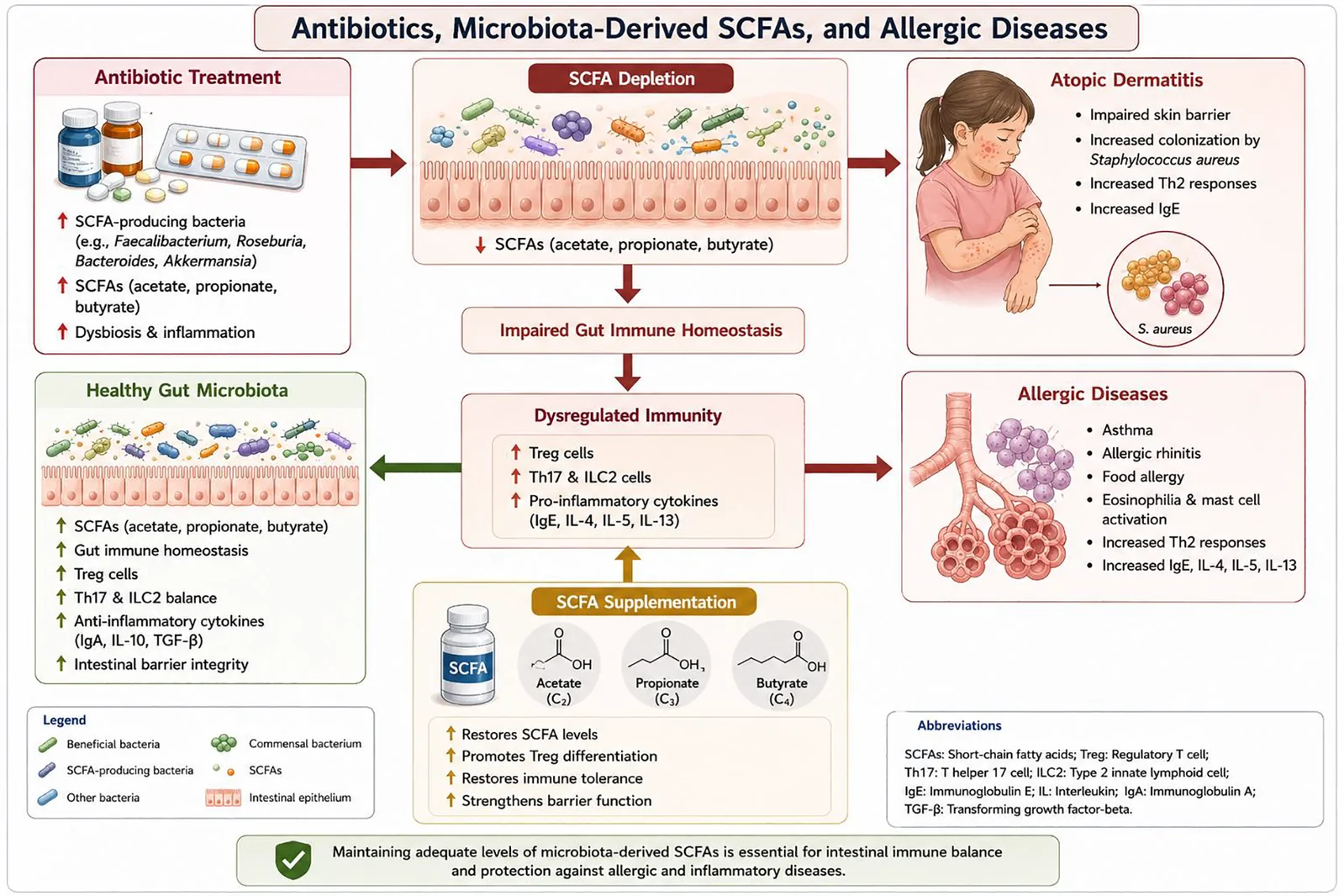
Antibiotic–microbial SCFA axis in allergic diseases. Antibiotic treatment disrupts the composition and metabolic activity of the gut microbiota, leading to a reduction in SCFA-producing bacteria, including members of the genera Faecalibacterium, Roseburia, Bacteroides, and Akkermansia. As a consequence, intestinal concentrations of the major microbiota-derived SCFAs—acetate, propionate, and butyrate—decline, resulting in impaired gut immune homeostasis and microbial dysbiosis. Reduced SCFA availability compromises intestinal barrier integrity and alters immune regulation, contributing to dysregulated immune responses characterized by diminished regulatory T-cell (Treg) activity, increased T helper 17 (Th17) and type 2 innate lymphoid cell (ILC2) responses, and enhanced production of pro-allergic cytokines and immunoglobulin E (IgE). SCFA depletion promotes the development and progression of allergic diseases by enhancing type 2 immune responses and inflammatory signaling pathways. In atopic dermatitis, reduced SCFA levels are associated with impaired skin barrier function, increased colonization by *Staphylococcus aureus*, elevated IgE production, and enhanced T helper 2 (Th2)-mediated inflammation. More broadly, dysregulated immunity contributes to the pathogenesis of allergic disorders, including asthma, allergic rhinitis, and food allergy, which are characterized by eosinophil and mast cell activation and increased levels of IL-4, IL-5, and IL-13. Conversely, a healthy gut microbiota maintains adequate SCFA production, promotes intestinal immune homeostasis, supports epithelial barrier integrity, enhances Treg-cell differentiation, and stimulates anti-inflammatory mediators such as interleukin-10 (IL-10), transforming growth factor-β (TGF-β), and immunoglobulin A (IgA). Supplementation with SCFAs, particularly butyrate, restores SCFA levels, strengthens epithelial barrier function, promotes immune tolerance, and counteracts allergic inflammation. Collectively, these findings highlight the critical role of microbiota-derived SCFAs in maintaining immune balance and protecting against allergic and inflammatory diseases.

The findings unveil a new cellular pathway that illustrates how selective gut dysbiosis, through the depletion of SCFA-producing bacteria by antibiotics, leads to allergic inflammation (**Figure 4**) (206). In line with findings that microbiota-derived metabolites are essential for immune modulation, Kabil et al. (206) showed that dietary supplementation with SCFAs in dysbiotic mice counters the effects of antibiotic-driven depletion of SCFA-producing bacteria, thus restoring normal immune homeostasis. They found that providing dietary SCFAs to dysbiotic mice can offset the immune disturbances caused by antibiotic-induced loss of SCFA-producing microbes and help re-establish immune balance. Although such supplementation does not correct the underlying microbial imbalance, it markedly suppresses ILC2 expansion as well as the downstream activation of B1 cells, basophils, mast cells, and eosinophils (206). This inhibitory effect persists even when exogenous IL-2 is administered to enhance IL-33–driven responses. These findings highlight important directions for future work exploring how SCFAs influence ILC2 development and transcriptional regulation. Multiple GPCRs are known to sense SCFAs, and several of these are present on ILC2, suggesting they may partially mediate SCFA-dependent regulation of ILC2 proliferation and activity (206). Moreover, Thio et al. (207) reported that butyrate—unlike acetate or propionate—suppresses IL-5 and IL-13 production by naïve lung ILC2 and limits their expansion during Alternaria alternata infection or IL-33–induced inflammation.

Early-life administration of broad-spectrum antibiotics has been described to promote susceptibility to Th17-mediated psoriasis and Th2-mediated Atopic dermatitis (AD) (208, 209). More importantly, an imbalance in gut bacterial homeostasis has been found as an important factor in the pathogenesis of atopic disorders (210, 211). The proposed mechanism is that gut epithelial inflammation caused by microbiota dysbiosis underlies chronic AD progression (211). Therefore, Kim et al. (212), by using an oral antibiotic cocktail-induced microbial depletion mouse model of AD, identified that intestinal microbial homeostasis plays a critical role in preventing the condition. Mechanistically, modulation of SCFA production by the gut microbiota regulated the balance between CD4+IL17+ T cells and CD4+FOXP3+ Treg cells, influencing the levels of ILC3 in the gut mucosa (212). Dysbiosis induced by antibiotic treatment caused intestinal inflammation and altered SCFA production. The balance between pro-inflammatory, Th17, and anti-inflammatory, Treg responses, which is crucial for gut immune homeostasis, is influenced by the composition of the commensal microbial community and its impact on the SCFA profile (213). Antibiotic treatment significantly downregulated the CD4+FOXP3+ Treg cells, which are known to maintain intestinal homeostasis (214). The intestinal IL17+/FOXP3+ Treg cell imbalance caused by microbiota alterations was rescued by fecal transplantation or oral administration of probiotics (212). These results suggest for the first time that an IL17+/CD4+FOXP3+ Treg cell imbalance in the gut could be a critical determinant for the development of AD in early life.

In order to preliminarily investigate the mechanism of vancomycin-induced gut microbiota dysbiosis in Allergic rhinitis (AR), Chen et al. (141) established a gut microbiota dysbiosis model induced by vancomycin in young mice and then established an Ovalbumin (OVA)-induced AR model in adult mice. The results showed that vancomycin caused the development of gut microbiota dysbiosis, primarily characterized by significantly decreased microbial diversity, including specific bacterial taxa. At the same time, the levels of serum OVA-specific Immunoglobulin E (IgE), total IgE, IL-4, and IL-5 were increased, and staining of nasal tissue showed exacerbated nasal inflammation (141). These findings suggest that gut microbiota dysbiosis promotes the dysregulation of Th2-type allergic responses in AR, which is consistent with previous studies on other allergic diseases. In the current work, Spearman correlation analysis showed that vancomycin aggravated AR by depleting specific bacterial taxa, including SCFA-producing bacteria. Notably, genera such as Ruminococcus and Lactobacillus were reduced in the vancomycin group and were positively correlated with fecal SCFA levels (141). The decline of Ruminococcus was associated with a significant decrease in fecal SCFAs, suggesting that vancomycin-induced gut dysbiosis may aggravate AR through SCFA depletion. Importantly, Chen et al. (141) showed for the first time in a mouse model of AR that butyrate supplementation significantly increased Foxp3 protein levels in the colon, which reflected enhanced differentiation of Tregs in the colonic mucosa. Treatment with butyrate increased serum IL-10 and Transforming Growth Factor beta 1 (TGF-β1) levels, thus enforcing immune tolerance and suppressing allergic responses. Such findings are also in line with previous data obtained in atopic dermatitis, where Treg balance in the colon attenuated allergic symptoms of this disease (212). Collectively, these findings suggest that impaired SCFA levels play a key role in vancomycin-induced gut dysbiosis and allergic inflammation, while butyrate may alleviate AR by promoting Treg-mediated immune regulation.

### 6.5 Antibiotic effect of microbiota SCFA imbalance in nervous system diseases

Antibiotic exposure profoundly alters gut microbiota composition, depleting key SCFA-producing bacteria. This results in a significant depletion of gut-derived metabolites that are critical for neuroimmune regulation, maintenance of BBB integrity, and gut-brain communication. Subsequently, antibiotic-driven SCFA depletion has emerged as one of the main mechanistic drivers for the development of a range of nervous system disorders. Lagamma et al. (215) showed that specific impairment of tonic and stress-induced epinephrine release via the sympathetic-adrenal medullary (SAM) system, without affecting glucocorticoid secretion via the hypothalamus-pituitary-adrenal (HPA) axis or parasympathetic responses (e.g., glucagon), follows gut microbiome depletion due to oral antibiotic treatment in young adult mice. These findings support the requirement for ongoing gut-brain axis interactions in peripheral, possibly central-catecholaminergic responses to acute hypoglycemic stress, both in Germ-free and conventionally colonized animals.

Although recolonization by cohousing with age-matched controls restored bacterial diversity and richness to control levels, the SAM stress axis remained hyporesponsive and was associated with decreased caecal butyrate, propionate, and acetate levels (215). While the exact mechanisms by which gut microbiota influence sympathoadrenal responses are not yet known, supplementation with bacterial SCFAs partially restored baseline and stress-induced epinephrine release even in the absence of microbiota-derived SCFAs. While several bacterial taxa significantly decreased in the antibiotic+ R cohort—belonging to Firmicutes and Bacteroidetes, known SCFA producers, such as Lachnospiraceae bacterium A4; Christensenella; Anaerotruncus sp. G3; Clostridium clostridioforme; Firmicutes bacterium ASF500; Bacteroides xylanisolvens; Bacteroides fragilis—were absent in the antibiotic-only group, implicating thereby these species and their SCFA metabolites in the attenuated SAM-specific neurohumoral response (215). In accordance with previous reports by Zarrinpar et al. (71), antibiotic treatment was associated with the complete disappearance of SCFAs in caecal samples, confirming successful gut sterilization. Acetate, propionate, and butyrate levels remained significantly lower compared to controls as far as two weeks after recolonization (antibiotic + R), highlighting the altered metabolic activity due to sustained changes in bacterial community composition (215). The addition of SCFAs to drinking water during antibiotic treatment (antibiotic + SCFA) reversed the impact of microbiome depletion as assayed using an intraperitoneal glucose tolerance test (IPGTT), normalizing glucose Area under the receiver operating characteristic (ROC) curve (AUC) values to those of control and SCFA-alone groups, suggesting a role for SCFAs in the recovery of glucose homeostasis (71). Furthermore, the supplementation with SCFAs significantly enhanced epinephrine responses to hypoglycemia, both in the SCFA-alone and antibiotic + SCFA groups. Collectively, these results reinforce the concept that SCFAs are small-molecule physiological mediators of the microbiota-gut-brain axis crosstalk that modulate not only aspects of the HPA axis (71).

In a study, Li et al. (168) used a paradigm of an adolescent rat model of stress-induced depression and assessed the protective effect of rifaximin on depression-like behavior through modulation of the gut microbiome, and explored related mechanisms. Chronic unpredictable mild stress (CUMS) disrupted both the overall gut microbiome composition and the levels of SCFAs in the brains of depressive rats. Furthermore, CUMS promoted the pro-inflammatory function of microglia and impeded neurodevelopment in adolescent rats (168). Li et al. (168) showed that rifaximin restored anti-inflammatory function to microglia and prevented abnormal microglial phagocytosis by modulating the gut microbiome and brain butyrate levels. Importantly, butyrate levels in the brains of CUMS rats significantly decreased, while rifaximin did not allow this decrease; moreover, rifaximin administered alone increased brain butyrate levels. Spearman correlation analysis showed associations of particular gut microbiota with SCFA levels, namely Ruminococcus bromii and Lachnospiraceae with butyrate (168). Further analysis showed that butyrate levels negatively correlated with TNF-α and IL-1 while positively correlating with IL-10 and IL-1β in the dentate gyrus, suggesting that butyrate is a key mediator of rifaximin’s regulatory effects on microglial function. Although previous studies have demonstrated the capability of sodium butyrate to modulate microglial inflammatory phenotypes, many such studies utilized super-physiological doses, questioning its physiological relevance. Based on the measured SCFA levels, Li et al. (168) sought to determine whether a physiological dose of sodium butyrate, 0.3 μM, under the influence of rifaximin, had the capacity to inhibit LPS-induced microglial activation *in vitro*. Accordingly, Sodium butyrate enhanced anti-inflammatory function in microglia and inhibited LPS-induced phagocytosis. Taken together, these findings support the hypothesis that butyrate mediates the protective effects of rifaximin on microglial function and thereby links gut microbiome modulation to improved neuroimmune homeostasis in stress-induced depression.

Rodenhouse et al. (216) found that major findings using the loss-of-function (antibiotic treatment) and gain-of-function (probiotics (PBX) treatment) models of gut microbiota include the following: antibiotic impaired functional recovery after Traumatic peripheral nerve injury (TPNI), while PBX rescued or improved post-injury functional recovery in the absence or presence of antibiotic, respectively. These findings, together with observations that antibiotics drastically depleted gut microbiota diversity and PBX restored microbial communities-particularly increasing the abundance of butyrate-producing bacteria-emphasize the crucial role of gut microbiome modulation in recovery after TPNI. Rodenhouse et al. (216) specifically noted increased abundance of butyrate-producing Ruminococcaceae in the 10-day PBX group and Bifidobacterium in the 10-day antibiotic-PBX -PBX group. Akkermansia was also significantly enriched in the 10-day PBX group and is known to produce butyrate, propionate, and acetate. Considering the systemic anti-inflammatory effects of SCFAs and the key role of macrophages in post-injury inflammation, these findings implicate SCFA-producing gut microbiota in improved functional recovery following TPNI and suggest that this may be through their immunomodulatory and anti-inflammatory effects.

Liang et al. (217) demonstrated that administration of cefazolin may directly exert anti-inflammatory effects and improve cognitive impairment caused by isoflurane anesthesia and exploratory laparotomy. In Luo et al. (167) study, they further investigated the impact of cefazolin on blood–brain barrier function and tight junction integrity in aged mice. Their results indicated that acetate, propionate, isobutyrate, and butyrate accounted for 76–93% of total SCFAs. Compared with controls, anesthesia and surgery alone, and cefazolin alone led to the reduction of total SCFAs and nearly all individual SCFAs, implying that SCFA depletion might be one cause of postoperative cognitive dysfunction (s) (167). Interestingly, perioperative cefazolin administration reversed the decline in total SCFAs, driven primarily by increased plasma acetate and propionate. These results suggest that modulation of SCFAs may underlie the protective effects of cefazolin against anesthesia- and surgery-induced cognitive impairments (167). There is evidence to suggest that SCFAs, and especially propionate, may modulate the function of the BBB. For instance, *in vitro* studies indicate that propionate binds FFAR3 on brain endothelium, triggers the Nuclear factor erythroid 2-related factor 2 (Nrf2) signaling pathway, and protects the BBB against oxidative stress (218, 219). Given that cefazolin improved BBB integrity, these findings support the idea that perioperative cefazolin may improve Postoperative cognitive dysfunction (POCD) by increasing plasma propionate and activating the FFAR3/Nrf2 pathway in brain endothelial cells.

### 6.6 Metabolic disorders

In particular, antibiotic use has been associated with increased metabolic disturbances, particularly when exposure occurs in early life (220, 221). Reijnders et al. (156) showed that, in obese humans, vancomycin treatment reduced the relative abundance of SCFA-producing bacteria along with a significantly reduced plasma and fecal SCFA concentration compared to placebo. SCFA fecal concentrations were significantly reduced after vancomycin, including but not limited to acetate, butyrate, caproate, and valerate, although in plasma, only butyrate tended to decrease after vancomycin, whereas no difference was evident after amoxicillin (156). KEGG annotation of gene expression data, for instance, indicated that vancomycin, and to a lesser extent amoxicillin, enhanced the expression of genes in antibiotic treatment participating in the PPAR signaling pathway and those coding for proteins associated with the mitochondrial Krebs cycle, fatty acid degradation, and other oxidative pathways, suggesting increased oxidative metabolism in antibiotic treatment. Besides, vancomycin reduced the expression of histone clustering genes. Similarly, vancomycin also downregulated gene sets associated with apoptosis, NF-κB, and adaptive and innate immunity, including Major Histocompatibility Complex (MHC)-I, T cell, B cell, and NK (natural killer) cell signaling genes. In contrast, genes regulating lysosomal breakdown were upregulated relative to placebo (156). A study has shown that azithromycin and florfenicol enhance adipogenesis in mice and alter the overall abundance, diversity, and composition of the gut microbiota and SCFA production (154). These antibiotics both reduced the colonic SCFA concentrations, but some differences in this change were found between the two, most likely reflecting antibiotic-specific changes in the relative abundance and number of SCFA-producing bacteria. Body fat percentage was significantly higher and SCFA content significantly lower in both antibiotic-treated groups compared to the controls (154).

The lower concentration of SCFAs may lead to obesity by several means. First, SCFAs not only provide energy but are also essential nutrients for intestinal cells and play a critical role in maintaining intestinal homeostasis. Therefore, a lack of SCFAs could cause nutritional deficiency in intestinal cells, reduce barrier defense, and increase susceptibility to low-grade inflammation, which promotes obesity (222). Second, some SCFAs, such as butyrate and propionate, inhibit obesity by stimulating gluconeogenesis through the cyclic AMP pathway and via brain-gut neural circuits and by inhibiting fat deposition in adipose tissue (223, 224). Thus, the lower production of these SCFAs could downregulate the related metabolic pathways and promote obesity. In recent years, dysbiosis of the gut microbiota has been implicated in metabolic diseases like T2D and obesity (115). In obese mice, changes in the gut microbiota led to metabolic endotoxemia and parameters associated with obesity, all of which were reduced after gut microbiota modulation by antibiotics. SCFA levels, together with changes in the microbial community composition, played an important role in obesity. In addition, it has been reported that supplementation of a high-fat diet with SCFAs protects against diet-induced obesity via a PPAR-dependent mechanism (115). By creating a model to evaluate adiposity, Cho et al. (155) demonstrated that a variety of sub-therapeutic antibiotic therapy (STAT) regimens alter the adiposity of post-weaning C57BL/6J mice. They hypothesize that STAT exposures select for a microbiota with increased metabolic activity, which extracts a greater percentage of calories from dietary complex carbohydrates that are relatively non-digestible and non-absorbable by control mice. The increased SCFA concentrations are metabolic products of this activity, then delivered in larger quantities via the portal circulation to the liver, promoting enhanced lipogenesis. Enhanced caloric absorption has been implicated as a mechanism for increased weight gain in other murine models of obesity. The observed increase in adiposity is consistent across several different STAT exposures, despite the use of different antibiotics (155). Their analyses of intestine, liver, and adipose tissue reveal that the primary effect of antibiotics is on the microbiota, which in turn affects downstream liver metabolism, while the adipose tissue passively accumulates the increased lipid load resulting from these upstream activities (155).

### 6.7 Cancer

Microbiota-derived metabolites have been shown to affect tumor growth in several models, and recent findings have extended this to breast cancer (BrCa) as well (225). For example, cadaverine treatment in mice with 4T1 BrCa tumors improved outcomes, with smaller tumors and less metastasis. In addition, cadaverine production was also diminished in BrCa patients and correlated with survival (226). To further explore this in BrCa models, Mckee et al. (225) performed fecal metabolomic analysis by Nuclear magnetic resonance (NMR) and observed significant metabolic dysregulation in the microbiota of vancomycin-treated animals, notably perturbations in the SCFAs acetate and butyrate. Gut microbiota-derived butyrate is readily absorbed and has been implicated in multiple diseases, including cancer, through its ability to inhibit HDACs. HDAC inhibition by butyrate has been demonstrated to sensitize cancer cells to ROS-induced apoptosis (227). *In vitro*, exogenous butyrate suppresses BrCa cell proliferation through the induction of senescence and apoptosis (228). Although Mckee et al. (225) transcriptomic data revealed no significant dysregulation in the transcription of cell cycle regulatory genes, they noted a decrease in the expression of pro-apoptotic genes like BNIP3 and increased expression of pro-survival genes such as HERPUD1 and URI, consistent with butyrate’s bioactivity. To enhance the clinical relevance of findings, Mckee et al. (225) utilized a BrCa-relevant antibiotic regimen. Animals were administered Cephalexin (8.64 mg/kg), an antibiotic commonly prescribed to BrCa patients prior to mastectomy in the US, and this treatment significantly enhanced tumor growth. Microbiota profiling revealed profound changes in microbial composition post-Cephalexin treatment. The genus most changed in both treatment groups, relative to pretreatment samples, was Lactobacillus; however, no significant differences between treatments were identified, suggesting the loss of Lactobacillus was more likely due to cage effects, tumor-microbiota interactions, or natural maturation of the microbiome, rather than administration of Cephalexin (229, 230). Of the genera significantly reduced compared with controls, several are known butyrate producers, such as Odoribacter and Anaerotruncus, or have genes necessary for butyrate production, such as Faecalibaculum and Alistipes (231, 232). Although butyrate produced by the microbiota has been shown to inhibit colorectal cancer cell proliferation *in vitro*, studies of tumor tissue suggest a paradoxical effect *in vivo* (233). Moreover, Lin et al. (164) analyzed the clinical profiles and fecal SCFA content of antibiotic-treated hematological malignancy (HM) patients, and they found that the incidence of AAD in these patients was 18.94%, influenced by multiple factors, including the duration and type of antibiotic administered, as well as serum albumin levels at the time of consultation. To further investigate, an AAD model was established in rats. SCFAs were found to attenuate clinical symptoms, enhance intestinal barrier function, and alleviate intestinal inflammation by modulating the gut microbiota (164). Intestinal epithelial cells maintain epithelial integrity and tight junctions through proteins like ZO-1 (234). The number of mucus-secreting goblet cells was decreased in AAD rats, but this effect was reversed by SCFA treatment. Moreover, SCFAs enhanced the expression of ZO-1 and CLDN3, improved mucosal barrier function, and decreased Plasmalemma vesicle associated protein (PLVAP) expression and intestinal permeability, as previously reported (235, 236).

### 6.8 CVD

Antibiotic-induced gut microbiota alteration has been shown to have either beneficial or adverse effects on cardiovascular disease, including hypertension, based on the type of antibiotic and the genetic background of the host (237). Hsu et al. (160) found that remodeling of the gut microbiome during critical phases in early development modifies later-life blood pressure. Administration of minocycline during pregnancy and lactation elevated offspring blood pressure, which was associated with reduced levels of acetate and butyrate, increased expression of SCFA receptors, and abnormal activation of the renin-angiotensin system (RAS) (160). Acetate has vasodilatory properties, and its oral addition counteracts hypertension programmed by maternal high-fat intake (238). The plasma levels of isobutyric acid and isovaleric acid are inversely related to blood pressure; thus, it could be postulated that any increases in these SCFAs serve more as counterbalancing rather than as causative agents in high-fructose diet (HF)-programmed hypertension (160).

Minocycline therapy lowered plasma levels of acetate and butyrate by potentially reducing populations of acetate-producing bacteria, including Lactobacillus, and inhibiting butyrate-producing microbes, such as the class Bacilli and genus Ruminococcus (239). Acetate and butyrate serve as ligands for GPR41, which has blood pressure-lowering properties (240). However, this vasodilation role of GPR41 may be inhibited through the induction of vasoconstriction in hypertension mediated by GPR43 and Olfr78 (241). Of note, minocycline treatment significantly augmented the expression of GPR43 and Olfr78 in both NDM and HF and minocycline administration (HFM) offspring. Thus, the hypertensive effects programmed by minocycline could be a consequence of its modification of SCFA levels and their receptors, shifting the balance of vascular tone toward vasoconstriction. Another study by Hsu et al. (242) found new insights into the mechanisms underlying minocycline-induced hypertension and the protective effects of acetate supplementation, focusing on gut microbiota and renal function that are summarized as follows: (1) Adult offspring developed hypertension at 12 weeks of age from dams exposed to minocycline during gestation and lactation; (2) maternal acetate supplementation prevented the development of hypertension in these offspring; (3) minocycline exposure during pregnancy and lactation lowered plasma acetic acid levels in offspring, restored by maternal acetate supplementation; (4) acetate supplementation increased the expression of the SCFA receptor GPR41 in offspring kidneys; (5) co-administration of minocycline and acetate led to a significant reduction in inflammatory cytokine levels in the kidneys and altered gut microbiota composition; and (6) protection against minocycline-induced hypertension with acetate supplementation was associated with increased abundances of the genera Roseburia, Bifidobacterium, and Coprococcus. Also, the genus Barnesiella, a lactate-producing bacterium, showed a decrease with acetate supplementation. Overall, maternal acetate supplementation helped restore the balance of acetate and SCFA-producing bacteria, therefore preventing developmental programming of hypertension.

The results of Ho et al. (159) indicate that targeted disruption of gut microbiota by antibiotic treatment can modulate circulating butyrate levels and influence susceptibility to neointimal hyperplasia in a rat model of arterial injury. Serum butyrate was reduced after treatment with vancomycin, indicating the presence of residual butyrate derived either from microbes not eliminated by vancomycin or from the diet (159). This reduction is consistent with the observed relative decrease in the Gram-positive Firmicutes phylum, targeted by vancomycin, and an increase in the Gram-negative Bacteroidetes phylum. Butyrate has been previously demonstrated to exert anti-proliferative and anti-migratory effects on VSMCs (243, 244). With the observed correlation of higher serum butyrate levels with reduced neointimal hyperplasia, these findings together support a direct action of butyrate on the inhibition of VSMC proliferation and migration for protection against neointimal hyperplasia.

## 7 SCFA as a potential biomarker in antibiotic therapy

Several analytical techniques have been used for the determination of SCFAs, including Gas chromatography (GC), GC–mass spectrometry (GC–MS), High-performance liquid chromatography (HPLC), liquid chromatography (LC)–MS, and stable isotope tracer methods (245). In Zhao et al. (245) study, a simple, accurate, robust GC method for the analysis of fecal SCFAs was established, and the changes in fecal SCFA profiles in rats induced by antibiotics were determined. Two commonly used broad-spectrum antibiotics, an advanced-generation cephalosporin, cefdinir, and a macrolide, azithromycin, were chosen. These results indicated that either cefdinir or azithromycin treatments significantly reduced all the SCFAs measured, and most did not return to baseline levels within 8 days after the withdrawal of the antibiotic treatments (245). Such findings have suggested that fecal SCFA may be a useful clinical marker guiding the use of antibiotics. As of today, there is limited research that systematically investigates antibiotic effects on fecal SCFAs (246). Very recent work in preterm infants has demonstrated that changes in microbiota are associated with alterations in SCFA levels; these can further be modulated by perinatal antibiotic exposures, such as maternal administration of penicillin, ampicillin, or ampicillin plus erythromycin (247, 248). Zhao et al. (245) data clearly showed that these two commonly used antimicrobials, cefdinir and azithromycin, strongly affected the SCFA composition, thus indicating SCFAs as sensitive markers of gut microbiota for antibiotic actions.

During the period of antibiotic administration, the fecal SCFAs AUC was significantly lower in the treated groups than in controls, excluding those of isobutyric and hexanoic acid (245). The effect of cefdinir on SCFAs was stronger than that of azithromycin. During the post-administration period, the AUCs of acetate, propionate, butyrate, and valeric acid remained distinctly lower in the cefdinir group than in controls, although they were higher compared with the period of antibiotic treatment. The AUC of isobutyric acid significantly increased during recovery, while that of isovaleric acid returned to a value comparable to that of the control group, indicating a rapid recovery of branched chain fatty acid (BCAAs)–producing bacteria. In the azithromycin-treated group, the AUCs of acetate, propionate, isobutyric acid, butyrate, isovaleric acid, and valeric acid remained distinctly lower, while hexanoic acid was the only SCFA whose AUC recovered within 8 days (245). These differences likely reflect the distinct antimicrobial spectra and activities of cefdinir and azithromycin (249, 250). SCFAs in the intestine have also been reported to inhibit the growth of pathogenic or opportunistic bacteria, thus providing protection against infection and inflammation (251, 252). The loss of both total and beneficial gut bacteria after antibiotic treatment can lead to reduced diversity and abundance of SCFA in feces. It is already known that antibiotics greatly disrupt the richness and diversity of the gut microbial community, which likely contributes to changes in SCFA levels (245). The clinical implications of the GC method developed in this study may further entail that SCFA profiles can act as personalized indices of drug safety and efficacy. In Gao et al. (253) study, shifts in microbial metabolism correlated well with changes in microbiota composition, suggesting that microbial SCFAs could be used as biomarkers for intestinal microbiota stability after antibiotic treatment. In fecal samples, the overall SCFAs and individual SCFAs-acetate, butyrate, and valerate-would be useful markers of gut health because fecal collection is easy. Previous studies have found that SCFAs can serve as non-invasive, valid, and reliable biomarkers in distinguishing between healthy subjects and patients suffering from intestinal microbial dysbiosis (254). Changes in microbial metabolites, such as SCFAs, provide a practical way of assessing the effect of antibiotics on intestinal microbiota. Notably, antibiotic treatment affected the ileum microbiota more promptly compared to fecal microbiota, which indicates the importance of examining the small intestinal microbiota for detecting early impacts of antibiotic interventions on gut homeostasis (253). Ampicillin, gentamicin, and metronidazole treatments lowered the concentration of acetate, with an earlier onset in the ileum from day 2 than in feces from day 7. This decrease probably reflects the loss of acetate-producing bacteria such as Bifidobacterium, Megasphaera, and Clostridium species (253). Furthermore, correlation analysis indicated that there was a positive correlation between the abundance of Ruminococcus and levels of butyrate in feces, suggesting a contribution of declining Ruminococcus to reduced butyrate in feces. Taken together, AGM treatment caused clear temporal changes in SCFAs, indicating that microbial carbohydrate fermentation was decreased in the gut.

## 8 Restoration strategies for antibiotic-associated SCFA imbalance

Generally, the levels of SCFAs are reduced during antibiotic therapy, so it is necessary to explore whether SCFA restoration during antibiotic treatment can alleviate related problems (**Table 3**). Evidence for probiotic-mediated modulation of intestinal SCFAs has been demonstrated in both human and animal studies. In a human trial, Lactobacillus plantarum 299V prevented the decline of SCFA levels during metronidazole treatment (278). In mice, Lactobacillus rhamnosus GG was as effective as the butyrate derivative tributyrin in preventing antibiotic-induced intestinal injury and in maintaining levels of the SCFA receptor GPR109a and transporter SLC5A8 (279). Because Lactobacilli lack the metabolic pathways to produce butyrate directly, the protective effects of L. rhamnosus GG likely result from cross-feeding interactions with the gut microbiota that increase luminal butyrate or through butyrate-independent mechanisms. Similarly, compounds secreted by Lactobacillus acidophilus ATCC4537 were shown to prevent enteropathogenic E. coli from inhibiting butyrate uptake in Caco-2 cells by preventing endocytosis of the monocarboxylate transporter isoform 1 (MCT1) (280).

**Table 3:** Restoration strategies for antibiotic-associated SCFA imbalance.

Restoration strategy	Study setting	Disease/condition	Antibiotic (type/dose/exposure)	Affected bacterial dysbiosis & SCFA disruption	Outputs after restoration	Reference
Lactobacillus strains (L. plantarum KLDS 1.0386, L. acidophilus KLDS 1.0901, and mixed culture)	*In vivo* (mouse model)	Antibiotic-associated diarrhea (AAD)	Ampicillin-induced diarrhea (dose/time not specified in excerpt; ampicillin exposure sufficient to induce AAD)	↓ Bacteroidetes; ↑ Escherichia–Shigella; disruption of SCFA-producing taxa; impaired microbial-SCFA synthesis pathways; reduced SCFA levels	Lactobacillus restored microbial diversity; increased SCFA production; recovered SCFA-associated pathways; inhibited TLR4/NF-κB signaling; ↓ pro-inflammatory cytokines; ↑ anti-inflammatory cytokines; increased tight junction proteins & aquaporins; alleviated intestinal inflammation & barrier damage	(255)
Lactobacillus casei	*In vivo* (Wistar rats)	Anti-TB drug-induced intestinal adverse reactions	Isoniazid + Rifampicin (daily for 42 days)	↓ Operational taxonomic units (OTUs) and diversity; ↓ Bacteroides, Akkermansia, Blautia; ↑ Lachnospiraceae NK4A136 group, Ruminococcus UCG-005; ↓ SCFAs (butyrate, valeric, hexanoic acids)	Lactobacillus casei restored microbial diversity and beneficial taxa; ↑ hexanoic acid; improved goblet cells, mucin-2, and intestinal barrier	(195, 256)
Lacticaseibacillus strains: L. paracasei CNCM I-1518 & I-3689, L. rhamnosus CNCM I-3690	Randomized, controlled trial, adults	H. pylori eradication therapy-induced gut dysbiosis	Pantoprazole, clarithromycin, and amoxicin (14 days)	↓ alpha-diversity; ↑ Candida; ↓ SCFAs; ↑ Enterobacteriaceae (Escherichia-Shigella, Klebsiella)	Faster recovery of microbiota beta-diversity; ↑ major SCFAs & valerate; ↓ potentially pathogenic Enterobacteriaceae; detection of viable ingested strains	(257)
Lactiplantibacillus plantarum ELF051	*In vivo* (mouse model)	AAD	500 mg/kg amoxicillin, 400 mg/kg clindamycin, and350 mg/kg streptomycin (14 days)	Dysbiosis; ↓ SCFAs; ↑ pro-inflammatory cytokines; impaired gut microbiota diversity	↑ SCFAs; ↑ gut bacterial diversity & abundance; ↓ IL-1β, TNF-α; ↑ IL-10; modulation of TLR4/MyD88/NF-κB & PI3K/AKT/NF-κB pathways; restoration of gut microbiota	(258)
Escherichia coli and butyrate producer Anaerostipes caccae	*In vivo* (mouse model)	Sorbitol intolerance	Streptomycin (5 days)	↓ Clostridia; impaired microbial sorbitol catabolism; gut dysbiosis	E. coli restored sorbitol catabolism but not Clostridia abundance; A. caccae restored Clostridia abundance via butyrate → activation of PPAR-γ, restoring epithelial hypoxia and protecting against sorbitol-induced diarrhea	(259)
Bifidobacterium animalis subsp. lactis BB-12	Randomized controlled trial in humans	AAD	Amoxicillin/clavulanate (875 mg), 7 days	↓ Gut microbiota diversity (Shannon index), ↓ SCFA production (acetate)	BB-12 yogurt mitigated antibiotic-induced reduction in fecal acetate, accelerated microbiota recovery, stabilized taxonomic profile over 30 days	(260)
Lactobacillus GG (LGG) ± Tributyrin	*In vivo* (C57BL/6 mice)	AAD	Metronidazole, neomycin sulfate, and vancomycin (7 days)	↓ Butyrate transporter and receptor expression, ↓ Na^+^/H^+^ exchanger, ↓ Cl⁻/HCO_3_⁻ exchanger, ↓ water channel proteins; disrupted gut barrier	LGG and/or tributyrin restored intestinal mRNA and protein expression of butyrate transporter/receptor, tight junctions, and ion/water transporters; prevented AAD	(261)
Clostridium butyricum/Butyrate supplementation	*In vivo* (vancomycin-treated mice)	Attenuated humoral immunity after rabies vaccination	Vancomycin (0.5 g/L) 4 weeks	↓ Butyrate-producing bacteria; ↓ SCFA levels; impaired humoral immunity (↓ IgM, IgG, VNAs); ↓ GC B cells and plasma cells	↑ SCFAs restored gut metabolites; ↑ IgM, IgG, VNAs; ↑ antigen-specific CD4+ T cells; ↑ germinal center B cells; ↑ plasma cell and antibody-secreting cell generation; Akt-mTOR pathway activation	(262)
Tributyrin (TB) supplementation	*In vivo* (C57BL/6 mice)	Gut dysbiosis	Ceftriaxone sodium, 400 mg/mL, gavage twice daily for 7 days	↓ α-diversity, ↓ SCFA-producing bacteria (Muribaculaceae, Bifidobacterium), ↑ potentially pathogenic bacteria (Bacteroidetes, Enterococcus)	↑ α-diversity, ↑ SCFA-producing bacteria, restored SCFA production, ↑ tight junction proteins (ZO-1, Occludin, MUC2), ↓ serum LPS and inflammatory cytokines (TNF-α, IL-6, IL-1β), suppression of NLRP3 inflammasome; low-dose TB more effective than high-dose	(263)
Butyrate	*In vivo* (C57BL/6J mice)	Ethanol consumption behavior	0.5 mg/mL bacitracin, 2.0 mg/mL neomycin, and 0.2 mg/mL vancomycin (2 weeks)	↓ butyrate-producing bacteria; gut microbiota dysbiosis	Prevented antibiotic-induced increase in ethanol intake; ↓ ethanol preference; microbiota diversity/composition largely unchanged; SCFA-mediated modulation of gut-brain axis	(264)
SCFA supplementation	*In vivo* (mice)	Innate anxiety	ampicillin (1 g/L), vancomycin (0.5 g/L), neomycin (1 g/L), and metronidazole (0.5 g/L) 3–4 weeks	Microbiota depletion; ↓ SCFA levels; altered anxiety-like behavior	SCFA administration restored innate anxiety responses; effects independent of subdiaphragmatic vagus nerve; modulation of gut-brain axis via SCFA	(265)
Butyrate supplementation	*In vivo* (mouse model)	Clostridium difficile-induced colitis	Clindamycin (7 days)	↓ Butyrate levels; intestinal barrier dysfunction; ↑ intestinal inflammation; ↑ epithelial permeability	↑ Butyrate restored tight junction expression; ↑ HIF-1 activation; ↓ intestinal inflammation; ↓ epithelial permeability; improved barrier function; mitigated colitis pathology	(266)
Sodium butyrate supplementation	*In vivo* (C57BL/6J mice)	Neuroinflammation	ABX cocktail consisted of 0.5 mg/mL bacitracin, 2.0 mg/mL neomycin, and 0.2 mg/mL vancomycin	ABX + ethanol ↑ MCP-1, TNF-α, IL-1β, IL-6, IL-10; ↑ microglial activation (Iba-1 (267)+ cells); ↓ GFAP+ astrocytes; microbiota depletion → ↓ endogenous butyrate	Sodium butyrate prevented ABX+ethanol-induced ↑ brain cytokines; prevented microglial activation and astrocyte loss; modulated neuroinflammatory pathways; prevented increase in ethanol consumption	(267)
Butyrate supplementation (maternal diet)	*In vivo* (maternal nonobese diabetic (NOD) mice & female offspring)	Type 1 diabetes (T1D) in female offspring	Vancomycin in drinking water during gestation and nursing (oral exposure; dose not specified)	Maternal gut dysbiosis; impaired SCFA-mediated immune tolerance	Butyrate restored SCFA signaling; inhibited maternal pro-inflammatory cytokine secretion; reduced recruitment of IFN-γ+ T cells in offspring pancreas; inhibited pancreatic DC activation; dampened T1D progression in female offspring	(193)
Maternal acetate supplementation	*In vivo* (rat)	Blood Pressure	Minocycline 50 mg/kg/day (gestation to lactation); acetate 200 mmol/L in drinking water	Maternal dysbiosis: ↓ plasma acetate; altered gut microbiota composition; ↓ SCFA receptor GPR41; reduced beneficial genera (Roseburia, Bifidobacterium, Coprococcus)	Acetate supplementation restored plasma acetate; increased GPR41 expression; increased SCFA-producing genera; protected offspring against minocycline-induced hypertension; improved gut microbiota composition	(242)
Polyphenol-rich grape powder	*In vivo*	Colitis, colitis-associated colon cancer (CAC)	Ampicillin, neomycin,metronidazole and vancomycin	Colitis and CAC: ↓ bacterial evenness; ↓ butyrate-producing Lachnospiraceae; decreased fecal butyrate; microbiota dysbiosis; antibiotic treatment prevented grape powder effects	10% grape powder restored gut bacterial evenness; increased relative abundance of butyrate-producing Lachnospiraceae; enhanced fecal butyrate; mitigated colitis and CAC in conventional mice; no effect in antibiotic-treated mice	(268)
Panax quinquefolius polysaccharides (WQP)	*In vivo* (rat model)	AAD	750 mg Lincomycin (5 days)	↓ Microbiota diversity; ↓ Lactobacillus, Bacteroides; ↑ Blautia, Coprococcus; dysregulated microbial community; ↓ SCFAs	↑ Lactobacillus, ↑ Bacteroides; ↓ Blautia, Coprococcus; ↑ acetate & propionate; improved microbiota structure; ↑ Occludin & Claudin-1; ↓ IL-1β, IL-6, IL-17A, TNF-α; ↑ IL-4 & IL-10; restored intestinal barrier	(269)
Cereus sinensis polysaccharide (CSP-1)	*In vivo* (C57BL/6 mice)	AAD	3 g Lincomycin (3 days)	↓ Microbial richness & diversity; ↑ Parabacteroides, Sutterella, Coprobacillus; ↓ beneficial taxa (Phasecolarctobacterium, Bifidobacterium); ↓ SCFAs; ↑ IL-1β, IL-2, TNF-α; impaired intestinal barrier	↑ Diversity & richness; ↑ Phasecolarctobacterium & Bifidobacterium; ↓ harmful taxa; ↑ SCFAs (via GC-MS); ↓ IL-1β, IL-2, TNF-α; improved barrier integrity; improved diarrhea score, water intake, body weight	(270)
Salidroside supplementation	*In vivo* (C57BL/6J mice)	Antibiotic-induced gut microbiota disturbance	200 mg ceftriaxone (3 days)	↓ Richness & diversity; ↓ Lactobacillus, Bifidobacterium; ↑ disease-associated taxa; ↓ SCFA levels; ↑ inflammation (TNF-α, IL-6); intestinal damage	↑ Microbiota richness & diversity; ↑ Lactobacillus & Bifidobacterium; ↓ pathogenic taxa; ↑ SCFA levels; improved barrier function; ↓ TNF-α & IL-6; low dose most effective for rapid SCFA & probiotic restoration	(271)
Dictyophora indusiata water-insoluble polysaccharides (DIPY)	*In vivo* (C57BL/6J mouse model)	AAD	3 g/kg lincomycin (3 days)	↓ Microbial diversity; ↓ Shannon & ACE indices; ↓ Parasutterella, Blautia; ↓ SCFAs (notably acetic acid); ↑ inflammatory mediators (LPS, MCP-1, TNF-α, IL-6)	DIPY ↑ microbial diversity; ↑ Shannon & ACE indices (p < 0.001); ↑ Parasutterella and Blautia; ↑ acetic acid levels (p < 0.001); ↓ LPS, MCP-1, TNF-α, IL-6; improved cecal morphology; attenuated AAD	(272)
Fiber-based enteral nutrition (soy + oat fiber)	Human randomized controlled pilot trial	Sepsis patients	β-lactam/β-lactamase inhibitorcombinations, carbapenems, cephalosporins, fluoroquinolones,and clindamycin; 24 hours	↓ SCFA-producing taxa at baseline; antibiotic exposure depresses SCFA producers and SCFA levels; risk of MDRO colonization due to loss of fermenters	Fiber group received median 32.1 g fiber by Day 3; trends toward ↑ SCFA-producing taxa (+61% vs –46% in control); ↑ fecal SCFA concentration (707 vs 118 µg/g); effects not statistically significant due to small sample; indicates potential for fiber to restore SCFA metabolism	(273)
Dietary nobiletin (NOB)	*In vivo* (mouse model)	Cefuroxime- and levofloxacin-induced gut dysbiosis & intestinal barrier dysfunction	Cefuroxime 12.5 g/L + Levofloxacin 10 g/L, 0.2 mL/day, 10 days	↓ Beneficial bacteria (Lachnospiraceae); ↓ SCFAs; intestinal barrier damage; ↑ LPS, TNF-α, IL-1β; goblet cell reduction	↑ Lachnospiraceae; ↑ butyrate; ↑ lithocholic acid; ↑ tight junction proteins; ↑ goblet cells; ↓ LPS, TNF-α, IL-1β; restored intestinal barrier and SCFA metabolism	(274)
Fecal Microbiota Transplantation (FMT)	Pediatric cohort, n = 9 rCDI patients; healthy controls n = 19	Recurrent Clostridioides difficile infection (rCDI)	Erythromycin	SCFA depletion: ↓ acetate, butyrate, propionate, isovalerate; underlying dysbiosis	Post-FMT (up to 12 months): ↑ acetate, propionate, isovalerate; combined SCFA levels restored; metabolite levels approached healthy controls	(275)
FMT	Clinical trial	Cirrhosis	5 days	↓ microbial diversity; ↓ autochthonous taxa; ↓ SCFAs; altered bile acid deconjugation & 7α-dehydroxylation	FMT restored microbial diversity, SCFA levels, bile acid metabolism, and functional microbial networks to baseline; no change in SOC group; well tolerated	(276)
FMT	*In vivo* (mouse)	Sepsis	Vancomycin 1.5 g/70kg andimipenem 2 g/70kg (4 days)	↑ Proteobacteria; ↓ Firmicutes; ↑ Escherichia–Shigella; ↓ Lactobacillus; impaired SCFA-mediated gut functions	FMT + SCFAs restored Firmicutes, Proteobacteria, Lactobacillus, and Escherichia–Shigella to near-normal levels; ↑ Occludin; ↓ NLRP3 & GSDMD-N; ↓ IL-1β & IL-18; inhibited pyroptosis; improved survival and reduced systemic inflammation	(277)

↑, Increase; ↓, Decrease.

Probiotics can directly and indirectly impact intestinal SCFAs. They themselves produce organic acids, such as acetate, and create an environment that facilitates SCFA production by bacteria. For example, *in vivo* production of acetate by the probiotic Bifidobacterium in the gastrointestinal tract lowers the risk of infection with enteropathogenic E. coli (281). Organic acid production by probiotics through their metabolism may lower luminal pH and oxygen levels and provide substrates for the synthesis of butyrate and propionate by other bacteria in the gut (282). Additionally, the growth of probiotics can reduce the concentration of undigested carbohydrates in the intestines, hence lowering the risk of diarrhea arising due to osmotic imbalance (283). Supplementation with butyrate-producing bacteria in the current study enhanced rabies virus (RABV)-specific antibody and virus-neutralizing antibody (VNA) responses in vancomycin-treated mice by promoting the generation of germinal center (GC) B cells, plasma cells (PCs), and RABV-specific antibody-secreting cells (ASCs) (262). In harmony with these findings, previous studies have shown that SCFA supplementation in mice treated with broad-spectrum antibiotics enhances trivalent influenza vaccine (TIV)-specific IgG production, indicating that SCFAs can broadly promote vaccine-induced humoral immunity and are not specific to RABV (284). Notably, these studies also reported that SCFA supplementation in mice on a normal diet did not enhance TIV immunogenicity, thus suggesting that SCFAs increase humoral responses mainly under conditions of host deficiency.

Mechanistically, SCFAs upregulate BAFF and ALDH1a2 expression in DCs, which then enhance the IgA and IgG production in B cells, improving the antibody responses generated by cholera toxin, as well as rescuing overall humoral responses in antibiotic-treated mice (285). Low concentrations of SCFAs (10 mM acetate or 1 mM butyrate) promote the production of pro-inflammatory cytokines that are important for inducing rapid Th1 responses, including IL-6, TNF-α, and granulocyte-macrophage colony-stimulating factor (GM-CSF), and chemokines such as C-X-C Motif Chemokine Ligand (CXCL)-1 and CXCL-10 (286). In primary B cells isolated from vancomycin-treated mice, butyrate elevated Blimp-1 expression through enhancement of mitochondrial function and activation of the Akt–mTOR pathway, thus enhancing the generation of CD138^+^ PCs. SCFAs have been shown to favor host antibody production and improve both intestinal IgA and systemic IgG through improvement of mitochondrial quality and fatty acid oxidation (FAO) (287). Indeed, high doses of SCFAs have also been reported to suppress antibody production through the regulation of AID and Blimp-1 expression, suggesting the dose-dependent effects of SCFAs on humoral immunity (288). Altogether, these data indicate that SCFAs increase the immunogenicity of rabies vaccines in antibiotic-treated mice.

Restoration of the Firmicutes/Bacteroidetes (F/B) ratio contributes to the reestablishment of microbial diversity and functional recovery of the gut microbiota, including enhanced production of SCFAs, improved gut barrier integrity, and modulation of immune responses (289). These effects are essential for counteracting the negative consequences of dysbiosis and maintaining overall gut and systemic health. Polyphenols, including catechins, quercetin, and baicalin, have been shown to alleviate dysbiosis by restoring microbial diversity and composition toward normal levels. They increase the relative abundance of beneficial genera, including Lachnospiraceae, Ruminococcaceae, and Akkermansia, while reducing pro-inflammatory taxa such as Anaeroplasma in a dose-dependent manner (290). Triterpenoid saponins, represented by ginsenoside Rk3, restore the F/B ratio by reducing Firmicutes and enhancing Bacteroidetes, while increasing the abundance of beneficial genera, including Anaerostipes, Alloprevotella, and Bacteroides (291). Other glycosides, such as salidroside, exhibit very impressive potential in correcting dysbiosis by increasing the abundance of beneficial bacteria, including Bacteroides, Lactobacillus, and Bifidobacterium, while decreasing harmful taxa, such as Helicobacter and Ruminococcus torques group. In general, the ability of these compounds to restore gut microbiota, inhibit pro-inflammatory responses, enhance gut barrier function, and stimulate SCFA production has been found to be consistently observed in compounds from different classes (289). Since the pharmacological activities are multifunctional, these compounds are ideal drug candidates for the management of antibiotic-induced dysbiosis and associated complications.

It has been shown that a quercetin-enriched diet recovered gut microbiota composition and structure in antibiotic-treated mice, showing a beneficial prebiotic-like effect (292). In Mi et al. (293) study, metagenomic analysis confirmed the above-mentioned effects on microbial structure. Of note, they found increased relative abundance of butyrate-producing bacterial species such as *F. rodentium*, *E. caecimuris*, *E. mucosicola*, *A. muciniphila*, and *R. hominis*, which are all beneficial for host health. *F. rodentium* is a bacterium in Erysipelotrichaceae family with strong fermentative abilities, enhancing carbohydrate metabolism, producing SCFAs, and having anticancer and anti-obesity properties (293). These observations underscore that quercetin promotes the growth of beneficial bacterial species while suppressing pathogenic ones, thereby restoring a healthy gut microbial structure. A recent study showed that reconstitution of butyrate exacerbated intestinal epithelial cell junction integrity and protected mice from graft-versus-host disease (294). Protection was independent of T-cell modulation, but rather involved limiting IEC damage caused by allo–human leukocyte antigen (HLA)-reactive T cells, which supports a role of butyrate in enhancing epithelial resistance to injury. Fachi et al. (266) similarly found a butyrate-mediated increase in Tregs and tolerogenic CD103^+^ DCs. In their model of CDI, butyrate administration increased Hif1a expression and stability, leading to upregulation of HIF-1 target genes *in vivo* (266). Importantly, the protective effect of butyrate was absent in Hif1a-deficient mice, with no improvement in intestinal permeability or bacterial translocation and no effects on clinical outcomes. Moreover, there was no additive benefit of butyrate in mice with constitutive activation of HIF-1α (VhlΔIEC mice), confirming that the protective actions of butyrate are dependent upon HIF-1α signaling in the IECs.

Tributyrin (TB) is a butyrate precursor, a major SCFA that exerts various beneficial effects on health (295). Yang et al. (263) results highlighted that supplementation with TB increased butyrate and other SCFAs, such as acetate and propionate, with greater efficacy for low-dose TB than high-dose TB. The beneficial effect of TB on SCFAs was probably a result of the improved delivery of butyrate to the intestinal tract and/or by increasing the populations of SCFA-producing bacteria, such as Muribaculaceae, Bifidobacterium, and Parabacteroides, able to produce other SCFAs (263). Following antibiotic intervention, mice exhibited a decrease in α-diversity, weight loss, diarrhea, an increase in cecal contents, and changes in colon length, reflecting perturbation of the gut microbiota (296). Loss of microbial diversity and abundance slows digestion and reduces feeding behavior, resulting in weight loss (297). A reduction in gut microbiota decreases intestinal peristalsis and excretion, resulting in retention of liquid and an increase in cecal volume, while diarrhea is an adverse symptom most commonly encountered during antibiotic use (298, 299). Improvement in all these symptoms after TB supplementation showed significantly better potency of low-dose TB over high-dose TB. TB intervention also increased the number of intestinal goblet cells and enhanced the expression of MUC2. Tight junction proteins, including ZO-1 and Occludin, were significantly upregulated, with low-dose TB again showing superior effects (263). Previous studies indicate that physiological concentrations of IL-1β and TNF-α increase intestinal epithelial tight junction permeability, and inhibiting IL-1β-mediated permeability protects against DSS-induced intestinal inflammation (300, 301). Thus, low-dose TB may protect intestinal barrier integrity by inhibiting IL-1β expression and preserving tight junction function.

## 9 Conclusion

Despite being the most effective therapy, antibiotic abuse, in concert with the remarkable ability of bacteria to evolve resistance rapidly, has resulted in the dismal situation where the emergence of antibiotic resistance and tolerance is increasingly common and thus impairs the treatment of infectious diseases. Importantly, antibiotic therapy places a high and generally underestimated metabolic cost on the host by disturbing the gut microbiota–SCFA axis. The reduction of key SCFAs, including acetate, propionate, and butyrate, triggers a set of physiological changes that range from defective epithelial energy metabolism and compromised mucosal barrier integrity to impaired immune tolerance, disturbed redox homeostasis, and disturbed gut–brain interaction. Altogether, these dysregulations contribute to enhanced inflammatory responses, heightened susceptibility to pathogens, metabolic derangement, and late-stage health complications. The identification of these hidden costs is essential for the development of safer therapeutic approaches and advancing a more holistic approach to antibiotic management.

Future studies need to focus on unraveling the complex manner in which different antibiotics reshape microbial metabolic function and SCFA production. Each antibiotic imposes a unique selective ecological pressure on the gut microbiota, and defining these specific metabolic fingerprints will be a key prerequisite for predicting outcomes in the host. Although many important mechanistic insights-FFAR2/FFAR3 signaling, PPARγ-regulated oxygen consumption, SCFA-driven HDAC inhibition-have been established using animal models, validation in humans remains an important next step. Another critical direction is more precise strategies for restoring SCFA-producing bacteria following antibiotic intervention. Novel microbiota-based therapies, such as tailored probiotics, engineered microbial consortia, and targeted synbiotics, may provide more predictable restitution of functional microbial communities. Direct supplementation of SCFA also deserves further investigation, including formulation and optimal dosing, as well as long-term safety. Parallel to these, there is a potential interaction of dietary patterns with antibiotics as an innovative way to prevent loss of SCFA, especially fiber-rich or metabolite-supportive diets. Finally, because early-life exposure to antibiotics can have long-lasting consequences on the maturation of the microbiota and SCFA dynamics, longitudinal cohorts following infants into adulthood are essential for defining windows of vulnerability and intervention opportunities.
